# Development of Melting-Curve-Based Real-Time PCR for Differentiating Medically Important *Candida* Species

**DOI:** 10.3390/ijms26199411

**Published:** 2025-09-26

**Authors:** Eliandro Reis Tavares, Virginia Prezzi Santos, Isabela Madeira de Castro, Paulo Henrique Guilherme Borges, Gislaine Silva-Rodrigues, Anna Paula Silva Olak, Guilherme Bartolomeu-Gonçalves, Jussevania Pereira Santos, Alexandre Tadachi Morey, Kelly Ishida, Sueli Fumie Yamada-Ogatta, Lucy Megumi Yamauchi

**Affiliations:** 1 Departamento de Microbiologia, Centro de Ciências Biológicas, Universidade Estadual de Londrina, Londrina CEP 86057-970, PR, Brazil; jussevania2302@gmail.com (J.P.S.); ogatta@uel.br (S.F.Y.-O.); 2 Escola de Medicina, Pontifícia Universidade Católica do Paraná, Londrina CEP 86067-000, PR, Brazil; 3 Programa de Pós-Graduação em Microbiologia, Departamento de Microbiologia, Centro de Ciências Biológicas, Universidade Estadual de Londrina, Londrina CEP 86057-970, PR, Brazil; virginiaprezzi@gmail.com (V.P.S.); isabela.mcastro@uel.br (I.M.d.C.); pauloguilhermeph@gmail.com (P.H.G.B.); gislaine.srodrigues@uel.br (G.S.-R.); 4 Programa de Pós-Graduação em Fisiopatologia Clínica e Laboratorial, Centro de Ciências da Saúde, Universidade Estadual de Londrina, Londrina CEP 86038-440, PR, Brazil; annapaula.olak@uel.br (A.P.S.O.); guilherme.bartolomeu@uel.br (G.B.-G.); 5 Instituto Federal de Educação, Ciência e Tecnologia do Rio Grande do Sul, Campus Canoas, Canoas CEP 92412-240, RS, Brazil; atmorey@gmail.com; 6 Laboratório de Quimioterapia Antifúngica, Instituto de Ciências Biológicas, Universidade de São Paulo, São Paulo CEP 05508-000, SP, Brazil; ishidakelly@usp.br

**Keywords:** intergenic spacer, ITS1, ribosomal DNA, molecular identification, SYBR Green

## Abstract

*Candida* species are the primary fungal pathogens of invasive infections associated with high morbidity and mortality. The identification of these microorganisms is critical for therapeutic management and control of hospital infection. Herein, assays targeting the Intergenic Spacer 2 (IGS2) and Internal Transcribed Spacer 1 (ITS1) from the rDNA locus were developed to differentiate *Candida* species. Based on consensus nucleotide sequences, specific primers and positive controls were designed, and standard PCR and real-time PCR (qPCR) assays were performed. All primers resulted in specific amplification of the molecular targets of each species with no amplifications of the negative template control. Furthermore, the primers were highly specific when tested with a range of fungal DNAs and no cross-reactivity was observed among *Candida* species. The assays presented a limit of detection (LoD) of 10 copies of positive control per reaction for all specific primers designed. Overall, our results showed that qPCR assays employing primers targeting the regions IGS2 and ITS1 completely differentiated between *Candida albicans*, *Candida auris*, *Candida parapsilosis*, *Candida tropicalis*, and *Nakazeomyces glabratus*, with great accuracy and no amplification of DNA from other fungal species.

## 1. Introduction

Rapid diagnosis of *Candida* infections is crucial. Studies have shown that in-patients subjected to a delayed fluconazole treatment following the onset of the initial signs are more likely to experience fatal outcomes [1]. Therefore, efficient diagnoses to avoid postponing an appropriate antifungal therapy are highly significant [1].

Healthy individuals may harbor *Candida* as a member of their microbiome. However, under certain conditions, this ordinarily harmless microorganism can act as an opportunistic pathogen, leading to infections. In fact, such opportunistic fungi can lead to serious health problems, particularly in immunocompromised individuals, underscoring the urgent need for effective prevention and treatment strategies [2,3,4,5]. In recent years, the incidence of candidemia has been steadily increasing in hospital settings around the world, which highlights the growing concern over its impact on vulnerable populations [6,7,8,9]. The risk factors include broad-spectrum antibiotic and immunosuppressive therapy, stem cell or solid organ transplantation, prolonged stays in intensive care units (ICUs), multiple invasive medical procedures, and parenteral nutrition [10,11,12,13].

Among the fungal species that cause human infections, approximately 80% of cases are attributed to *C. albicans*, followed by non-*albicans Candida* (NAC) species. Worldwide, approximately 2,000,000 new cases of oral candidiasis, 1,300,000 of esophageal candidiasis, and approximately 134,000,000 of recurrent vulvovaginal candidiasis are reported yearly. Regarding severe candidiasis (bloodstream infections and invasive candidiasis), approximately 1,565,000 new cases are estimated, with 995,000 deaths (63.6%). Particularly in bloodstream infection, approximately 939,000 have negative results in culture-based identification [14]. In Brazil, the most common NAC species are *Candida parapsilosis*, *Candida tropicalis*, *Nakaseomyces glabratus* (formerly known as *Candida glabrata*), *Pichia kudriavzevii* (formerly known as *Candida krusei*), *Candida auris*, and *Candida dubliniensis* [15].

Although *C. albicans* is the predominant species, a significant increase in the isolation of other NAC species as causative agents of candidiasis has been reported. For instance, *C. tropicalis* is the leading cause of infection in neutropenic individuals and in those patients staying in ICUs. In addition, *C. tropicalis* is associated with high mortality rates, ranging from 55 to 60% in adults and 26 to 40% in pediatric patients [16]. *N. glabratus* is associated with oral infections in the elderly and denture wearers, is the second most frequent causative agent of vulvovaginitis, and is related to cases of candidemia, especially in immunocompromised patients and in ICUs. Neonatal, transplant, and parenteral nutrition patients are at higher risk of infections by *C. parapsilosis*. *P. kudriavzevii*, in turn, is frequently isolated from patients undergoing stem cell transplantation, due to the use of azoles [17].

Traditional microbiological methods for identifying *Candida* species require several days and rely on the isolation of yeasts from different biological sources [18]. In fact, this process involves several steps, including the assessment of germ tube and chlamydospore formation, optimal growth temperatures, as well as the ability to utilize different carbon and nitrogen sources [18,19,20].

Several DNA-based methods have emerged as powerful tools for the detection of *Candida* species. Among these, PCR-based techniques enable rapid and accurate identification, significantly improving sensitivity and specificity [18,19,20,21,22]. Moreover, molecular techniques such as multi-locus sequence typing (MLST) provides a robust framework for analyzing genetic variations across multiple loci, enhancing the precision of species identification [23]. Similarly, randomly amplified polymorphic DNA (RAPD) employs a unique amplification process that reveals polymorphisms in DNA sequences, thus offering valuable insights into the genetic diversity of *Candida* strains [23]. Collectively, these DNA-based approaches represent a significant advancement in the diagnosis of candidiasis and contribute to more effective monitoring and treatment strategies.

Several PCR-based assays target the ribosomal RNA (rRNA) gene cluster, which plays a crucial role in the identification and differentiation of various microorganisms. This gene cluster comprises four essential ribosomal genes: 18S, 5.8S, and 25–28S, along with the 5S gene. Additionally, it includes the Internal Transcribed Spacers (ITSs) 1 and 2, as well as the Intergenic Spacers (IGSs) 1 and 2 [24,25]. The ITS regions are particularly remarkable due to their high conservation across *Candida* species, which has established them as reliable targets for a wide range of molecular applications. These regions can be explored using multiple approaches, such as ITS length polymorphism [26,27], DNA sequencing [28], restriction fragment length polymorphism [29], and DNA hybridization probes [29,30]. Furthermore, PCR followed by restriction fragment length polymorphism assays, targeting either IGS2 or the full-length IGS, has also been successfully utilized to differentiate closely related *Candida* species and other fungi [31,32]. Each of these methods investigates the genetic features of the rDNA gene cluster, providing a robust framework for research in the context of clinical diagnostics. These results indicate that these regions are reliable targets for detecting different *Candida* species.

Systems based on hydrolysis probes, such as TaqMan, are widely used for the detection of *Candida* spp., but may present some important issues. The use of fluorogenic probes specific to each species increases the cost of the method. In addition to the system itself being more expensive, it also requires thermocyclers with multiple detection channels. This may restrict the application of probe-based qPCR, especially in places with limited resources. In contrast, the melting curve analysis allows species to be differentiated based on their different melting temperatures (Tm), using only intercalating dyes such as SYBR Green [33], thus eliminating the need for probes and multi-channel equipment. This approach not only reduces operating costs, but also requires only one detection channel (green), which is present in most conventional real-time thermal cyclers.

Given the critical concerns regarding *Candida* infections, this study aimed to develop a specific and highly sensitive multiplex real-time PCR (m-qPCR) assay, based on melting curve analysis, designed for the accurate differentiation of five *Candida* species. These assays utilize primers specifically designed to target the IGS2 region in *C. albicans*, *C. tropicalis*, *C. parapsilosis*, and *N. glabratus* (formerly *C. glabrata*), and the ITS1 region in *C. auris*. This approach seeks to enhance diagnostic accuracy and facilitate rapid fungal identification within clinical laboratory settings.

## 2. Results

Initially, the IGS2 and ITS nucleotide sequences of *Candida* species and *C. auris*, respectively—available in the GenBank nucleotide database—were analyzed (Supplementary Figure S1), leading to the design of specific primer pairs for *C. albicans*, *C. auris*, *C. parapsilosis*, *C. tropicalis*, and *N. glabratus* (Table 1). In addition, a synthetic plasmid harboring all targets (positive control) was also designed (Supplementary Figure S2). Each primer pair was checked for similarity with other nucleotide sequences using the BLAST algorithm (v.2.15.0), and the results showed no matches other than the aforementioned *Candida* species. Furthermore, matches were not detected within the human genome. These results indicate that cross-reactivity with non-target sequences is unlikely to occur.

Following in silico analyses, monoplex PCR assays were performed using the positive control and 1 µM of each designed primer. These reactions successfully generated amplicons of the expected lengths: 303 bp for *C. albicans* and *C. tropicalis*, 240 bp for *C. auris*, 218 bp for *C. parapsilosis*, and 295 bp for *N. glabratus* (Table 1/Supplementary Figure S3). The annealing temperature was set at 62 °C to establish the appropriate amplification conditions for target detection.

Additionally, a second round of monoplex PCR assays was conducted using 100 ng of genomic DNA obtained from *C. albicans* ATCC 26790, *C. auris* CBS 10913, *C. parapsilosis* ATCC 1975, *C. tropicalis* ATCC 28707, and *N. glabratus* ATCC 518. The amplicons generated from these PCR runs matched the expected lengths, confirming the specificity of the primers designed in this study (Table 2).

**Figure 1:**
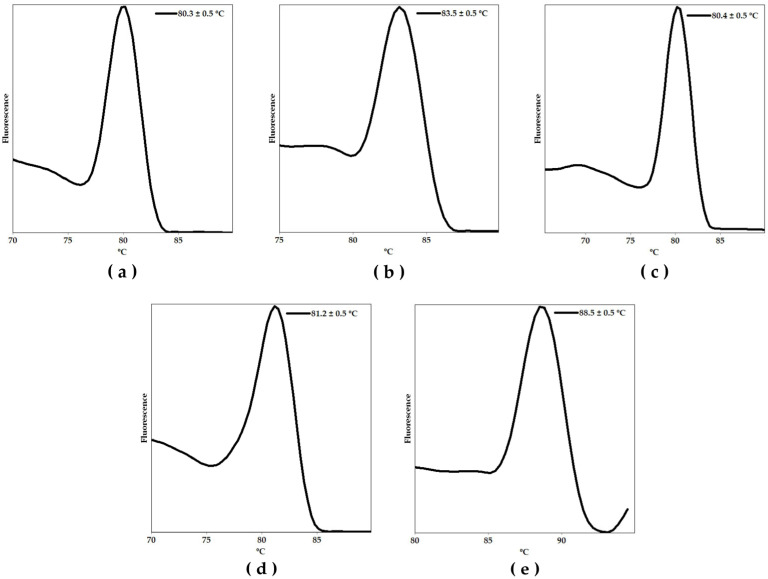
Melting curve analyses showing the melting temperature (Tm) peaks of the amplicons associated with the identification of (**a**) *Candida albicans*, (**b**) *Candida auris*, (**c**) *Candida parapsilosis*, (**d**) *Candida tropicalis*, and (**e**) *Nakaseomyces glabratus* amplicons using the positive (plasmid) controls harboring all targets.

Thereafter, we performed a monoplex qPCR assay using the synthetic positive control and 1 µM of each primer pair in a Rotor Gene Q-5Plex HRM platform to determine the melting temperature (Tm) peak of the specific amplicons. These results are shown in Figure 1a–e. Each graph represents the fluorescence signal as a function of temperature, showing the characteristic Tm of each specific amplicon. The observed Tm values were as follows: 80.3 ± 0.5 º C, 83.5 ± 0.5 °C, 80.4 ± 0.5 °C, 81.2 ± 0.5 °C, and 88.5 ± 0.5 °C, for *C. albicans*, *C. auris*, *C. parapsilosis*, *C. tropicalis*, and *N. glabratus*, respectively.

**Figure 2:**
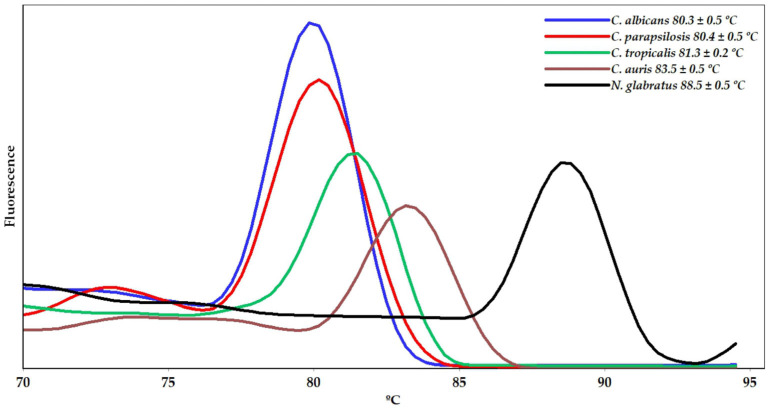
Melting curve analysis from qPCR assays performed individually in a single run showing the melting temperature (Tm) peaks of the amplicons corresponding to the identification of *Candida* and *Nakazeomyces* species.

Moreover, a qPCR assay using the primers for the species studied herein was carried out individually within a single run. The results, presented in Figure 2, showed melting peak temperatures equivalent to those observed in the monoplex qPCR system.

The analytical performance of the qPCR assays was also evaluated using DNA purified from cultures of *Candida* species. Amplification signals were detected for all yeasts tested, generating dissociation curves with single peaks, similar to those obtained with the positive controls.

To further assess primer specificity beyond in silico analysis, qPCR was performed using nucleic acids from a panel of bacterial and fungal organisms (Table 2). Amplification signals were exclusively observed for the targeted *Candida* species, thereby confirming the absence of cross-reactivity with non-target sequences.

**Figure 3:**
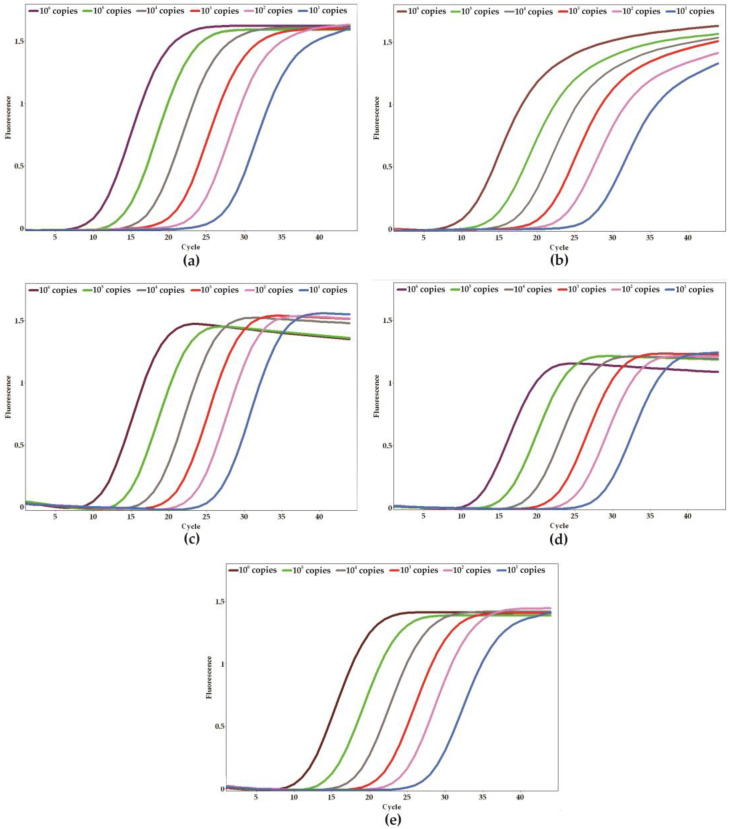
Analytical sensitivity of the qPCR system and the limit of detection for each specific target. (**a**) *Candida albicans*, (**b**) *Candida auris*, (**c**) *Candida parapsilosis*, (**d**) *Candida tropicalis*, and (**e**) *Nakaseomyces glabratus*. Amplification plot of serial dilutions resulting in 10^1^ to 10^6^ copies of the positive (plasmid) control harboring all targets.

**Figure 4:**
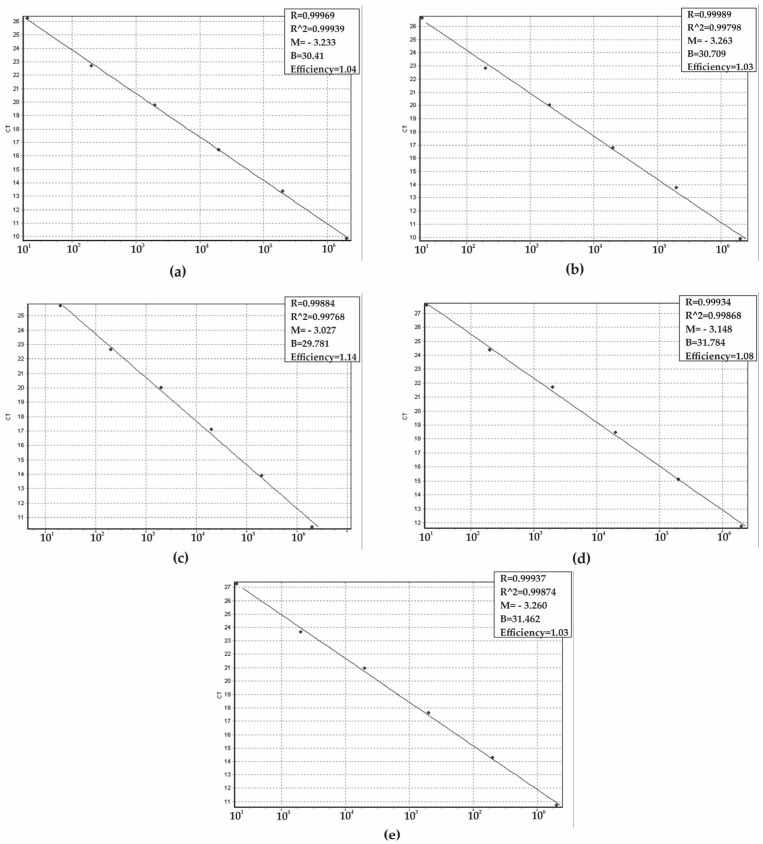
Standard curves obtained by linear regression of the threshold cycle (CT) versus copy numbers to determine the efficiency of the qPCR system. (**a**) *Candida albicans*, (**b**) *Candida auris*, (**c**) *Candida parapsilosis*, (**d**) *Candida tropicalis*, and (**e**) *Nakaseomyces glabratus.* Slope (M), regression coefficient (R), and efficiency of the real-time PCR method are shown (**a**–**e**).

To determine the analytical sensitivity of the qPCR assay and establish the limit of detection (LoD), a series of ten-fold serial dilutions of positive controls was carried out (ranging from 10^0^ to 10^6^ copies per reaction). The sensitivity assays were conducted in triplicate across five separate days, yielding a total of 15 replicates to ensure the robustness of the results. The LoD for each target gene was established at 10 copies per reaction (Figure 3a–e). The mean CT values for the target genes, which correspond to the smallest detectable quantities, were <26 for *C. parapsilosis*, <27 for *C. albicans* and *C. auris*, and <28 for *C. tropicalis* and *N. glabratus*. Furthermore, the efficiency of the reactions was assessed using the slopes of the standard curves, with values that ranged from 100% to 110% (Figure 4a–e).

To evaluate the quality of the nucleic acid purification and the presence of potential interfering substances, primers targeting the human tRNA processing ribonuclease P (RNase P) gene were also included in the qPCR, resulting in a Tm value of 82.5 ± 0.50 °C (Supplementary Figure S3).

**Figure 5:**
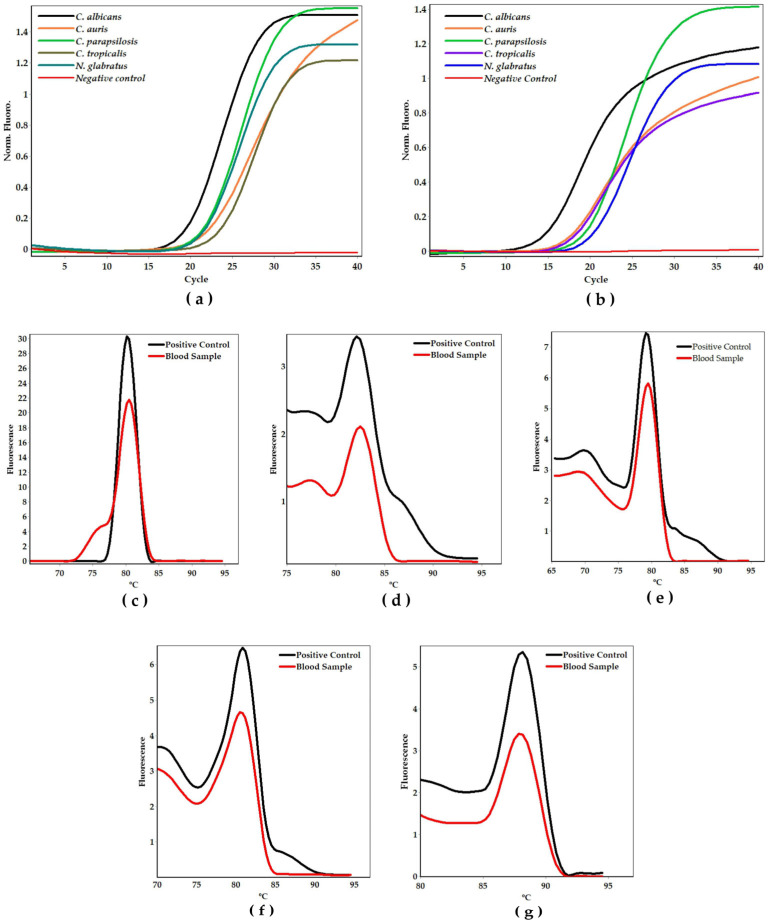
Amplification profiles of target genes using DNA obtained by the boiling method (**a**) and direct colony qPCR (**b**). Melting temperature peaks of *Candida albicans* (**c**), *Candida auris* (**d**), *Candida parapsilosis* (**e**), *Candida tropicalis* (**f**), and *Nakaseomyces glabratus* (**g**) amplicons using the blood samples spiked with each target *Candida* species.

To evaluate the effect of simplified DNA extraction methods on qPCR efficiency and turnaround time, genomic DNA obtained through the boiling method and colony qPCR was directly used in the assays (Figure 5a,b). The results were consistent with the amplification assays whose template DNA was purified using the phenol–chloroform DNA extraction method. Furthermore, the efficiency of the system for detecting yeasts from blood samples was assessed using the assay (Figure 5c–g), which resulted in characteristic amplification patterns for all primers corresponding to the species included in the study.

## 3. Discussion

Nucleic acid amplification tests (NAATs) have been widely used in clinical laboratories to detect etiological agents of infectious diseases, owing to their high sensitivity and specificity, as well as their ability to deliver rapid results [36]. In contrast, traditional culture-based tests for identifying microbial agents are often time-consuming, labor-intensive, and prone to displaying limited sensitivity [37].

In this study, a new method for detecting the most common causative species of candidiasis is presented and can be applied for both diagnosis and research. We utilized the IGS2 [*C. albicans*, *C. tropicalis*, *C. parapsilosis* and *N. glabratus* (formerly *C. glabrata*)] and ITS1 (*C. auris*) regions of ribosomal DNA genes for the development of a qPCR assay for the differentiation of five causative agents of candidiasis. Notably, the ITS, IGS, and NTS regions of ribosomal DNA genes have been widely used for the diagnosis and phylogenetic analysis of etiological agents of infectious diseases [38,39,40,41,42].

To ensure the specificity and accuracy of our assays, we meticulously selected target nucleotide sequences based on the following criteria: (i) gene essentiality; (ii) conserved sequences within the species to eliminate any risk of false-negative or false-positive results due to genetic variations; (iii) non-overlapping melting temperatures; and (iv) limited presence of secondary structures—such as homodimers and heterodimers—up to 10% of the Tm and total ΔG values of the primers.

The results shown in Table 1 represent a detailed view of the specific primers—CaNTS2, CauITS1, CpNTS2, CtNTS2, and NgNTS2—for the specific identification of *C*. *albicans*, *C*. *auris*, *C*. *parapsilosis*, *C*. *tropicalis*, and *N*. *glabratus*—using the NTS region. A crucial step relied on the amplification profile using CaITS1, specifically designed for *C. auris* identification. This specificity is extremely relevant, considering the emergence of antifungal resistance in this species, representing a significant challenge for public health [43,44,45]. Our findings confirmed that NgNTS2 led to specific amplification of *N. glabratus*, a pathogen that holds significant importance due to its antifungal resistance profile. Accurate identification of this pathogen is crucial, and it allows for the implementation of suitable therapeutic procedures for infection treatment, contributing to reducing the mortality and morbidity rates [46]. As for *C. parapsilosis*, using CpNTS2 primers, specific identification was also achieved. Considering the impact of this species on nosocomial infections, particularly among immunocompromised individuals, which rank just after *C. albicans*, it is imperative to ensure precise identification [47,48]. Finally, the assay using CtNTS2 demonstrated specificity for *C. tropicalis*. This species presents clinical relevance, due to the prevalence of systemic infections and azole resistance. Therefore, this highlights the necessity for the development of rapid methodologies for detection and monitoring of *C. tropicalis* [16,49].

Furthermore, no amplification was observed for fungal species other than those for which the primers were designed, including other yeasts and filamentous fungi. Amplifications were also not observed when bacterial DNA was used as a template in the assays. These results ensure the high specificity of the method proposed in this study, highlighting its methodological robustness.

The assay designed and optimized in our study offers numerous advantages, such as reduced time for accurate diagnosis with specific identification of the causative agents. The method is based on the use of an intercalating dye, which reduces the overall cost of qPCR [33], eliminating the need for additional probes and requiring only a single channel for fluorescence detection. Furthermore, the test can be performed using conventional PCR equipment and allows for simultaneous detection of multiple *Candida* species, in different reactions, and in a single run, resulting in significant savings in time and laboratory resources.

Dye-based qPCR assays coupled with the melting curve strategy to detect infectious microorganisms have been used in some studies. For example, Tavares et al. [42] used the IGS1 region of the ribosomal DNA (rDNA) as the target to differentiate *Cryptococcus gattii* sensu lato and *Cryptococcus neoformans* sensu lato using qPCR followed by melting curve analysis. Otaguiri et al. [50] proposed a melting-curve-based multiplex qPCR assay targeting the cfb gene to detect *Streptococcus agalactiae* in rectal–vaginal samples from pregnant women. Van Bergen et al. [51] implemented a melting-curve-based qPCR assay for the detection of *Plasmodium* in a routine clinical laboratory. Wan et al. [52] developed melting-curve-based RT-qPCR for the simultaneous detection of four human coronaviruses (HCoV-229E, HCoV-OC43, HCoV-NL63, and HCoV-HKU1). Additionally, Tavares et al. [53] developed melting-curve-based RT-qPCR for the simultaneous detection of SARS-CoV-2, Influenza A virus, Human Respiratory Syncytial Virus, and Human Rhinovirus B. The method led to efficient amplification of the mentioned viruses, with parameters of sensitivity and specificity similar to those of standard commercial tests.

Rapid and accurate detection and identification of *Candida* species are critical for timely treatment and optimal patient outcomes, as different species exhibit distinct antifungal susceptibility profiles that can directly influence therapeutic decisions. The results highlight that the simplified purification method maintains qPCR efficiency despite its procedural simplicity [54]. Thus, the use of the boiling method as a viable and low-cost alternative for DNA extraction in molecular protocols, and colony qPCR, may represent a relevant advance for clinical laboratories, especially in resource-limited settings [54]. 

Moreover, we assessed the qPCR performance using human blood samples artificially contaminated with different *Candida* species. Bloodstream candidiasis is commonly diagnosed through culture, which is considered the gold standard. However, the application of this method can directly impact prognosis and mortality, often delaying the initiation of optimized antifungal therapy, especially in infections caused by multidrug-resistant species such as *C. albicans* and *C. auris*. The concept of time to positivity (TTP) refers to the amount of time required for a culture to yield a positive result; *Candida* species exhibit slower growth rates compared to most bacterial species, prolonging TTP and influencing patient prognosis [55]. Therefore, the implementation of rapid methods for the identification of specific etiological agents directly from the bloodstream facilitates faster decision-making, ensures the selection of the appropriate antifungal therapy, and contributes to the effective management of infections caused by resistant *Candida* species.

A system that combines magnetic resonance with PCR [T2 Magnetic Resonance (T2MR) assay] to detect five species of *Candida*—*C. albicans*, *C. tropicalis*, *C. parapsilosis*, *P. kudriavzevii* (formerly *C. krusei*), and *N. glabratus*) [56]—was developed by T2 Biosystems and approved by the FDA (Food and Drug Administration). This test provides results within 3 to 5 h from bloodstream samples for routine clinical use. However, some estimates suggest that each test can cost up to USD 150, apart from the cost of the equipment [57]. Assuming that Brazil is classified as a developing country, these costs represent a significant barrier to the effective implementation of this method within the Brazilian healthcare system.

Despite the high specificity of the proposed method, we recognize some limitations inherent to the approach used. In conventional PCR, differentiation between *Candida albicans*, *C. tropicalis*, and *N. glabratus* can be challenging due to the similar size of the amplified fragments, requiring high-resolution electrophoretic analysis to avoid ambiguous interpretations. Similarly, in qPCR with SYBR Green, we observed that the melting profiles of *C. albicans* and *C. parapsilosis* exhibit highly similar dissociation temperatures, which can compromise the unequivocal distinction between these species when amplified simultaneously. To overcome this limitation, we propose that the reactions be performed individually in the same run, which maintains the simplicity and speed of the technique, in addition to preserving its applicability in laboratories with basic infrastructure. Finally, we acknowledge that the present study did not evaluate the performance of the method in clinical samples, since the main objective was to validate the approach using reference strains. Nevertheless, the results obtained demonstrate the potential of the methodology as an accessible and efficient tool for screening and preliminary identification of yeasts of medical importance, with future applicability in clinical contexts after additional validations.

In conclusion, *Candida* IGS2 and ITS2 primers were designed to specifically amplify the target of each species in a single PCR run. These reactions reduce the cost and time required for *Candida* identification due to their simplicity and low cost, facilitating their application in routine clinical laboratory testing. The *Candida* identification primers showed great sensitivity and specificity, allowing DNA amplification exclusively for the target species. Overall, this study showed that the designed specific primers were able to differentiate medically important yeasts, including *C. albicans*, *C. auris*, *C. tropicalis*, *C. parapsilosis*, and *N. glabratus*. Finally, these molecular tests for *Candida* identification represent a new, sensitive, and specific method that enhances diagnostic efficiency. This improvement is important to optimize the management of patients at risk of systemic candidiasis, as it contributes to the rapid and early diagnosis of these infections and supports cost-effective treatment strategies.

## 4. Materials and Methods

### 4.1. PCR Primers and Positive Control Design

The nucleotide sequences of the IGS2 region of the ribosomal DNA gene of *C. albicans* (12 sequences), *C. parapsilosis* (12 sequences), *C. tropicalis* (3 sequences), and *N. glabratus* (formerly *C. glabrata*) (47 sequences), and the ITS1 region of the ribosomal DNA gene of *C. auris* (50 sequences), were retrieved from the GenBank/EMBL databases (available at http://www.ncbi.nlm.nih.gov, accessed on 10 July 2025). All the sequencies were subjected to multiple alignments by the ClustalW tool using BioEdit software (v.7.2.0) (Supplementary Table S1) to obtain a consensus sequence for each species. Specific primers were designed based on the consensus sequences of each species using the PrimerQuest^TM^ and OligoAnalyzer^TM^ tools (both available at http://www.idtdna.com, accessed on 6 March 2023). Moreover, the other yeast species were included in the alignment—*C. dubliniensis*, *Debaryomyces hansenii* (formerly *Candida famata*), *Meyerozyma carpophila* (formerly *Candida carpophila*), *Pichia guilliermondii*, *Pichia caribbica*, *Pichia norvegensis*, *Candida zeylanoides*, and *Candida inconspicua.* To ensure specificity, the NTS2 and ITS1 sequences from each *Candida* species and *N. glabratus*, the primer sequences targeting the designated regions of the ribosomal DNA locus, were compared with nucleotide sequences available in the GenBank database of the National Center for Biotechnology Information (NCBI, http://www.ncbi.nlm.nih.gov, accessed on 10 July 2025) using the BLAST algorithm (blastn). Detailed information regarding the primer sequences, melting temperatures, and expected amplicon lengths is shown in Table 1. The amplicon sequences generated by each specific primer were inserted into the pUC57 plasmid to serve as a positive control (Supplementary Figure S1).

### 4.2. Microorganisms and Culture Conditions

A panel of 49 fungal and 8 bacterial strains (Table 2) was used for the development of the assay. Five colonies from each fungal and bacterial strain were inoculated into Sabouraud Dextrose Broth (SDB, Himedia, Thane, India) and Tryptic Soy Broth (TSB, Oxoid, São Paulo, Brazil), respectively. The microbial cultures were incubated at 37 °C for 48 h for fungi and 24 h for bacteria. Following incubation, the cells were harvested by centrifugation (10,000× *g*, 5 min), washed twice with sterile saline (NaCl 0.85%), and processed for DNA purification. Fungal and bacterial strains were stored at −80 °C, in SDB and TSB, respectively, and supplemented with 20% glycerol.

### 4.3. DNA Extraction

To extract genomic DNA, the cells were resuspended in a lysis buffer containing 10 mM Tris-HCl (pH 8.0), 2% Triton X-100, 1% SDS, 100 mM NaCl, and 1 mM EDTA, along with phenol solution (Sigma-Aldrich, St. Louis, MO, USA). Glass beads were added, and the mixture was vigorously stirred to disrupt cells. After centrifugation and recovery of the aqueous phase, the DNA was purified with a phenol:chloroform:isoamyl alcohol solution (25:24:1), followed by precipitation using a 2.5-fold volume of ice-cold absolute ethanol (J.T. Baker, NJ, USA). The resulting DNA pellet was washed with 70% ethanol, dried at room temperature, and resuspended in ultrapure water. DNA concentration was determined by absorbance at 260 nm using a spectrophotometer (Synergy HT, Biotek, VT, USA). After quantification, the genomic DNA of each isolate was adjusted to a concentration of 50 ng/µL.

### 4.4. PCR Design

The PCR conditions were determined through a two-step process. First, the designed oligonucleotides were tested in conventional PCR with a final volume of 20 μL containing 1× PCR buffer, 2.5 mM MgCl_2_, 1.25 µM of each dNTP, 0.65 U *Taq* DNA polymerase (Invitrogen, Waltham, MA, USA), 0.5 to 2 μM of each primer, and 1 × 10^6^ copies of the positive control, to establish optimal annealing temperatures and primer concentrations. The amplification reactions were performed in a Veriti^®^ 96-well Thermal Cycler (Applied Biosystems, Singapore) using an initial denaturation at 95 °C for 2 min. This was followed by 35 cycles, each consisting of 95 °C for 30 s (denaturation), 30 s at a temperature gradient from 60 °C to 70 °C (for optimal annealing temperature determination; Supplementary Figure S3), and 72 °C for 30 s (extension). A final extension step was carried out at 72 °C for 10 min. Reactions without nucleic acid templates were included as a negative template control (NTC). Afterwards, amplicons were analyzed in 2% agarose gel electrophoresis and stained with ethidium-bromide (0.5 µg/mL) (Sigma-Aldrich, St. Louis, MO, USA).

An annealing temperature of 62 °C and primer concentration of 1 μM, along with 100 ng of DNA from *C. albicans* ATCC26790, *C. auris* CBS 10913, *N. glabrata* ATCC518, *C. parapsilosis* ATCC1975, and *C. tropicalis* ATCC28707, were selected and used to validate the amplification conditions. Subsequently, all qPCR assays were performed in a Rotor Gene Q-5Plex HRM (Qiagen, NW, Germany) in a final volume of 20 µL containing 1 × 10^6^ copies of the positive control, and 1 µM each of oligonucleotide and QuantiNova SYBR Green PCR mix (Qiagen, NW, Germany), according to the manufacturer’s recommendation. The cycling profile was as follows: 2 min at 50 °C, initial denaturation at 95 °C for 10 min, followed by 45 cycles of 30 s at 95 °C, 30 s at 62 °C, and 30 s at 72 °C. Melting curves were acquired with an increase in temperature ranging from 60 to 99 °C, with a time holding of 60 s at 0.5 °C at each step. NTC reactions were carried out simultaneously. Data were analyzed using the Rotor-Gene Q series software version 2.1.0.9.

### 4.5. Analytical Specificity, Sensitivity, and Validation

To determine the sensitivity of the qPCR assay, positive controls ranging from 10^0^ to 10^6^ copies per reaction were used to create a standard curve for each primer pair, based on the CT values as a function of the plasmid copy number. All amplification reactions were executed in duplicate in three separate experiments. The number of plasmid copies was determined by dividing the total mass of plasmids by the individual mass of each plasmid copy, as proposed by Thermo Fisher Scientific (2008) [58], according to the equation *m* = (*n*).(1.096 × 10^−21^ g/bp) [where *m* = mass and *n* = size of the plasmid plus insert, in base pairs], thus obtaining the number of copies per μL. Furthermore, the R^2^ values were calculated to evaluate the efficiency of the reactions. The efficiency (E) of each reaction was determined using the slope of standard curves according to the following equation: E = 10^−1/slope^. Each assay was carried out in triplicate on five consecutive days. A quantity of 100 ng of genomic DNA purified from the microorganisms listed in Table 2 was used for specificity analyses.

### 4.6. Analytical Validation with DNA-Boiling Purification, Culture-qPCR and Blood Samples

An experiment was carried out to assess the amplification performance and the efficiency of the system using DNA obtained by boiling. *C. albicans* ATCC 26790, *C. auris* CBS 10913, *C. parapsilosis* ATCC 22019, *C. tropicalis* ATCC 28707, and *N. glabratus* ATCC 2001) were cultivated on Sabouraud Dextrose Agar (SDA) for 48 h at 37 °C. After cultivation, one colony-forming unit (CFU) of each species was collected and added to 100 µL of Phosphate Buffered Saline (PBS) and boiled for 10 min. Afterwards, the system was centrifuged at 14,000× *g*, and the supernatant was recovered. Five microliters of the recovered material were used for qPCR amplification, according to the conditions described above.

Also, a second colony was recovered from cultivation and submitted directly to amplification, following the same protocol of amplification as described above. Additionally, a third experiment was carried out to simulate the detection performance in blood samples. Whole blood samples (*n* = 3) were obtained from adult men; the sample collection was approved by the Research Ethics Committee of the State University of Londrina (CEP/UEL) under the document number 47784621.2.0000.5231 and approval number 4.862.243. The participants signed an informed consent form, expressing their agreement on the use of their samples and the publication of the results described herein. A quantity of 200 µL of human blood was artificially contaminated with one CFU of each cultured yeast and used to purify the total genomic DNA using the QIAamp^®^ DNA mini Kit (Qiagen) according to the manufacturer’s recommendations. The purified total DNA was used in the qPCR assays as previously described. Positive controls containing 10^6^ copies of the plasmid were used in three different assays, and reactions without the addition of template DNA were used as negative controls. All amplification reactions were carried out in duplicate and in three independent experiments.

## 5. Patents

This study resulted in a patent application to the Brazilian National Institute of Intellectual Property, deposit number BR 10 2024 015 279 4.

## Figures and Tables

**Table 1:** Characteristics of oligonucleotide primers utilized in the amplification assays.

Species	Target	Primers (5′ → 3′)		Amplicon Size (pb)	Annealing Temperature (°C)
*C. albicans*	IGS2	CaNTS2-F	GCCTCAATTTGAACGTGGTACTGC	302	62
		CaNTS2-R	TAGCCAAACCAACCATTACGGGTG		
*C. auris*	ITS1	CauITS1-F	GGATTTTAAAACTAACCCAACG	240	62
		CauITS1-R	TTTTGTGAATGCAACGCC		
*C. parapsilosis*	IGS2	CpNTS2-F	CCCTGATGCCACCAACACC	218	62
		CpNTS2-R	GCTAGAGCGTCGTTGTAAGAAG		
*C. tropicalis*	IGS2	CtNTS2-F	GGGCGTAGAATTCGATGGGAGTGA	302	62
		CtNTS2-R	GACACTTGGGAGGGGCTTACTAGAG		
*N. glabratus*	IGS2	NgNTS2-F	AGTACCCCCGGACCGAGCTT	295	62
		NgNTS2-R	CGTGCGACGGCACACGTTTT		
Ribonuclease P [34,35]		RNaseP-F	AGATTTGGACCTGCGAGCG	65	62
		RNaseP-R	GAGCGGCTGTCTCCACAAGT		

IGS, Intergenic Spacer; ITS, Internal Transcribed Spacer.

**Table 2:** Positive control and microorganisms used to assess the specificity of the melting-curve-based real-time PCR.

Strains/Isolates	Origin	CaNTS2	CauNTS2	CpNTS2	CtNTS2	NgNTS2
Positive control	Plasmid	+	+	+	+	+
*Candida albicans* ATCC 26790	Fiocruz ^a^	+	−	−	−	−
*C. albicans* ATCC 10231	Fiocruz ^a^	+	−	−	−	−
*C. albicans* ATCC 14053	BioMMLab ^b^	+	−	−	−	−
*C. albicans* 4	BioMMLab ^b^	+	−	−	−	−
*C. albicans* 5	BioMMLab ^b^	+	−	−	−	−
*C. albicans* 8	BioMMLab ^b^	+	−	−	−	−
*Candida auris* CBS 10913	LQA ^c^	−	+	−	−	−
*C. auris* CBS 12766	LQA ^c^	−	+	−	−	−
*Candida parapsilosis* ATCC 22019	Fiocruz ^a^	−	−	+	−	−
*C. parapsilosis* ATCC 1975	Fiocruz ^a^	−	−	+	−	−
*C. parapsilosis* 41	BioMMLab ^b^	−	−	+	−	−
*C. parapsilosis* 42	BioMMLab ^b^	−	−	+	−	−
*C. parapsilosis* 43	BioMMLab ^b^	−	−	+	−	−
*Candida tropicalis* ATCC 28707	Fiocruz ^a^	−	−	−	+	−
*C. tropicalis* 12	BioMMLab ^b^	−	−	−	+	−
*C. tropicalis* 13	BioMMLab ^b^	−	−	−	+	−
*C. tropicalis* 14	BioMMLab ^b^	−	−	−	+	−
*Nakaseomyces glabratus* * ATCC 2001	Fiocruz ^a^	−	−	−	−	+
*N. glabratus* ATCC 518	Fiocruz ^a^	−	−	−	−	+
*N. glabratus* 23	BioMMLab ^b^	−	−	−	−	+
*N. glabratus* 24	BioMMLab ^b^	−	−	−	−	+
*N. glabratus* 26	BioMMLab ^b^	−	−	−	−	+
*Candida bracarensis* PT1217	UMinho ^d^	−	−	−	−	−
*Candida dubliniensis* ATCC 974	Fiocruz ^a^	−	−	−	−	−
*C. dubliniensis* 83C	BioMMLab ^b^	−	−	−	−	−
*Candida guilliermondii* PT822	UMinho ^d^	−	−	−	−	−
*Candida kefyr* 1	BioMMLab ^b^	−	−	−	−	−
*Candida lusitaneae* PT1007	UMinho ^d^	−	−	−	−	−
*Candida metapsilosis* PT2263	UMinho ^d^	−	−	−	−	−
*Candida orthopsilosis* PT2259	UMinho ^d^	−	−	−	−	−
*Aspergillus flavus*	CMRP/UEL ^e^	−	−	−	−	−
*Aspergillus fumigatus*	CMRP/UEL ^e^	−	−	−	−	−
*Aspergillus niger*	CMRP/UEL ^e^	−	−	−	−	−
*Aspergillus terreus*	CMRP/UEL ^e^	−	−	−	−	−
*Cryptococcus gattii* ATCC 24065	Fiocruz ^a^	−	−	−	−	−
*C. gattii* ATCC 56990	Fiocruz ^a^	−	−	−	−	−
*C. gattii* ATCC 24066	Fiocruz ^a^	−	−	−	−	−
*Cryptococcus neoformans* ATCC 34872	Fiocruz ^a^	−	−	−	−	−
*C. neoformans* ATCC 66031	Fiocruz ^a^	−	−	−	−	−
*Hismiddlelasma capsulatum*	EJV/UEL ^f^	−	−	−	−	−
*Issatchenkia orientalis* ATCC 6258	BioMMLab ^b^	−	−	−	−	−
*Paracoccidiodes brasiliensis* 18	EJV/UEL ^f^	−	−	−	−	−
*Pichia kudriavzevii* ** ATCC 34135	Fiocruz ^a^	−	−	−	−	−
*P. kudriavzevii* ATCC 520	Fiocruz ^a^	−	−	−	−	−
*P. kudriavzevii* 36	BioMMLab ^b^	−	−	−	−	−
*P. kudriavzevii* 42	BioMMLab ^b^	−	−	−	−	−
*P. kudriavzevii* 39	BioMMLab ^b^	−	−	−	−	−
*Sporothrix* sp.	EJV/UEL ^f^	−	−	−	−	−
*Trichophyton rubrum*	EJV/UEL ^f^	−	−	−	−	−
*Enterococcus faecium* ATCC 6569	Fiocruz ^b^	−	−	−	−	−
*Enterococcus faecalis* ATCC 29212	Fiocruz ^b^	−	−	−	−	−
*Staphylococcus aureus* BEC 9393	Fiocruz ^b^	−	−	−	−	−
*S. aureus* MRSA PSA 598	BioMMLab ^b^	−	−	−	−	−
*S. aureus* MRSA PSA 149	BioMMLab ^b^	−	−	−	−	−
*Staphylococcus epidermidis* ATCC 35984	Fiocruz ^a^	−	−	−	−	−
*Staphylococcus haemolyticus* ATCC 29968	Fiocruz ^a^	−	−	−	−	−
*Pseudomonas aeroginosa* PA01	Fiocruz ^a^	−	−	−	−	−

^a^ Fiocruz, Fundação Oswaldo Cruz; ^b^ BioMMLab, Laboratório de Biologia Molecular de Microrganismo/Universidade Estadual de Londrina; ^c^ LQA, Laboratório de Quimioterapia Antifúngica/Instituto de Ciências Biológicas/Univerisade de São Paulo, ^d^ Universidade do Minho, Braga, Portugal; ^e^ CMRP/UEL, Coleção microbiológica rede paranaense/Universidade Estadual de Londrina; ^f^ EJV/UEL, Dr. E.J. Venancio-Laboratory/Universidade Estadual de Londrina. * *Nakaseomyces glabratus* (formerly *C. glabrata*); ** *Pichia kudriavzevii* (formerly *C. krusei*).

## Data Availability

Data are contained within the article and Supplementary Materials.

## Supplementary Materials

The following supporting information can be downloaded at: https://www.mdpi.com/article/10.3390/ijms26199411/s1.
