# Supplementary material for: Development of Melting-Curve-Based Real-Time PCR for Differentiating Medically Important Candida Species
Source: Int J Mol Sci. 2025 Sep 26;26(19):9411. doi: 10.3390/ijms26199411 (PMC12524715; doi:10.3390/ijms26199411)
Supplement: Supplementary file 1 [file ijms-26-09411-s001.zip › ijms-3708087-supplementary.pdf]

International Journal of *Molecular Sciences*

MDPI

Supplementary Table S1. Diagrammatic alignment representation of CaNTS2, CpNTS2, CtNTS2 and NgNTS2, forward and reverse primers, and *Candida albicans* (**a**), *C. parapsilosis* (**b**), *C. tropicalis* (**c**) and *Nakazeomyces glabratus* (**d**) NTS2 region (partial) of rDNA locus; and CauITS1, forward and reverse primers, and *C. auris* (**e**) ITS1 region (partial) rDNA locus. Computed by Multiple Sequence Alignment ClustalW algorithim.

(a) Diagrammatic alignment representation of CaNTS2 forward and reverse primers and *Candida albicans* NTS2 region (partial) of rDNA locus. Computed by Multiple Sequence Alignment ClustalW algorithim.

```
              ....|....| ....|....| ....|....| ....|....| ....|....| ....|....| ....|....| ....|....| ....|....|
                  5         15         25         35         45         55         65         75         85
FN554376.1    GAAAAAATAA AAAAATGCGA ACCTTATCTG CTGTCTCTCA CGTGACCAAA TTCACTTTTA GGGCTCTAGC CCCACAACCA CCAAAGTGTA
FN554375.1    GAAAAAATAA AAAAATGCGA ACCTTATCTG CTGTCTCTCA CGTGACCAAA TTCACTTTTA GGGCTCTAGC CCCACAACCA CCAAAGTGTA
CP128824.1    GAAAAAATAA AAAAATGCGA ACCTTATCTG CTGTCTCTCA CGTGACCAAA TTCACTTTTA GGGCTCTAGC CCCACAACCA CCAAAGTGTA
CP128824.1    GAAAAAATAA AAAAATGCGA ACCTTATCTG CTGTCTCTCA CGTGACCAAA TTCACTTTTA GGGCTCTAGC CCCACAACCA CCAAAGTGTA
CP128824.1    GAAAAAATAA AAAAATGCGA ACCTTATCTG CTGTCTCTCA CGTGACCAAA TTCACTTTTA GGGCTCTAGC CCCACAACCA CCAAAGTGTA
CP025165.1    GAAAAAATAA AAAAATGCGA ACCTTATCTG CTGTCTCTCA CGTGACCAAA TTCACTTTTA GGGCTCTAGC CCCACAACCA CCAAAGTGTA
CP025157.1    GAAAAAATAA AAAAATGCGA ACCTTATCTG CTGTCTCTCA CGTGACCAAA TTCACTTTTA GGGCTCTAGC CCCACAACCA CCAAAGTGTA
CP025182.1    GAAAAAATAA AAAAATGCGA ACCTTATCTG CTGTCTCTCA CGTGACCAAA TTCACTTTTA GGGCTCTAGC CCCACAACCA CCAAAGTGTA
CP025174.1    GAAAAAATAA AAAAATGCGA ACCTTATCTG CTGTCTCTCA CGTGACCAAA TTCACTTTTA GGGCTCTAGC CCCACAACCA CCAAAGTGTA
CP017630.1    GAAAAAATAA AAAAATGCGA ACCTTATCTG CTGTCTCTCA CGTGACCAAA TTCACTTTTA GGGCTCTAGC CCCACAACCA CCAAAGTGTA
CP032012.1    GAAAAAATAA AAAAATGCGA ACCTTATCTG CTGTCTCTCA CGTGACCAAA TTCACTTTTA GGGCTCTAGC CCCACAACCA CCAAAGTGTA
CP032012.1    GAAAAAATAA AAAAATGCGA ACCTTATCTG CTGTCTCTCA CGTGACCAAA TTCACTTTTA GGGCTCTAGC CCCACAACCA CCAAAGTGTA
CaNTS2-F      ---------- ---------- ---------- ---------- ---------- ---------- ---------- ---------- ----------
CaNTS2-R      ---------- ---------- ---------- ---------- ---------- ---------- ---------- ---------- ----------

              ....|....| ....|....| ....|....| ....|....| ....|....| ....|....| ....|....| ....|....| ....|....|
                  95        105        115        125        135        145        155        165        175
FN554376.1    TGTGCTGTCG CTGCAGGGGA GGGGTAATCA GGGTGCCCAA ATTATGTGGA GCCATTTTTT GAGCCGGAAA GTTGGGTGGC TGTGGCACAA
FN554375.1    TGTGCTGTCG CTGCAGGGGA GGGGTAATCA GGGTGCCCAA ATTATGTGGA GCCATTTTTT GAGCCGGAAA GTTGGGTGGC TGTGGCACAA
CP128824.1    TGTGCTGTCG CTGCAGGGGA GGGGTAATCA GGGTGCCCAA ATTATGTGGA GCCATTTTTT GAGCCGGAAA GTTGGGTGGC TGTGGCACAA
CP128824.1    TGTGCTGTCG CTGCAGGGGA GGGGTAATCG GGGTGCCCAA ATTGTGTGGA GCCATTTTTT GAGCCGGAAA GTTGGGTGGC TGTGGCACAA
CP128824.1    TGTGCTGTCG CTGCAGGGGA GGGGTAATCG GGGTGCCCAA ATTGTGTGGA GCCATTTTTT GAGCCGGAAA GTTGGGTGGC TGTGGCACAA
CP025165.1    TGTGCTGTCG CTGCAGGGGA GGGGTAATCG GGGTGCCCAA ATTGTGTGGA GCCATTTTTT GAGCCGGAAA GTTGGGTGGC TGTGGCACAA
CP025157.1    TGTGCTGTCG CTGCAGGGGA GGGGTAATCG GGGTGCCCAA ATTGTGTGGA GCCATTTTTT GAGCCGGAAA GTTGGGTGGC TGTGGCACAA
CP025182.1    TGTGCTGTCG CTGCAGGGGA GGGGTAATCG GGGTGCCCAA ATTGTGTGGA GCCATTTTTT GAGCCGGAAA GTTGGGTGGC TGTGGCACAA
CP025174.1    TGTGCTGTCG CTGCAGGGGA GGGGTAATCG GGGTGCCCAA ATTGTGTGGA GCCATTTTTT GAGCCGGAAA GTTGGGTGGC TGTGGCACAA
CP017630.1    TGTGCTGTCG CTGCAGGGGA GGGGTAATCG GGGTGCCCAA ATTGTGTGGA GCCATTTTTT GAGCCGGAAA GTTGGGTGGC TGTGGCACAA
CP032012.1    TGTGCTGTCG CTGCAGGGGA GGGGTAATCG GGGTGCCCAA ATTGTGTGGA GCCATTTTTT GAGCCGGAAA GTTGGGTGGC TGTGGCACAA
```

International Journal of
Molecular Sciences

MDPI

```
CP032012.1   TGTGCTGTCG CTGCAGGGGA GGGGTAATCG GGGTGCCCAA ATTGTGTGGA GCCATTTTTT GAGCCGGAAA GTTGGGTGGC TGTGGCACAA
CaNTS2-F     ---------- ---------- ---------- ---------- ---------- ---------- ---------- ---------- ----------
CaNTS2-R     ---------- ---------- ---------- ---------- ---------- ---------- ---------- ---------- ----------

             ....|....| ....|....| ....|....| ....|....| ....|....| ....|....| ....|....| ....|....| ....|....|
                 185        195        205        215        225        235        245        255        265
FN554376.1   AACGGGAATA TGTATGTTCG GGTGGCCAGT TGACTGGGTT GTAGCAGTGC TGCAGCTACG AATGTTAGAG ACAAAATGCA GTCCAGGACC
FN554375.1   AACGGGAATA TGTATGTTCG GGTGGCCAGT TGACTGGGTT GTAGCAGTGC TGCAGCTACG AATGTTAGAG ACAAAATGCA GTCCAGGACC
CP128824.1   AACGGGAATA TGTATGTCCG GGTGGCCAGT TGACTGGGTT GTAGCAGTGC TGCAGCTACG AATGTTAGAG ACAAAATGTA GTCCAGGGCC
CP128824.1   AACGGGAATA TGTATGTCCG GGTGGCCAGT TGACTGGGTT GTAGCAGTGC TGCAGCTACG AATGTTAGAG ACAAAATGTA GTCCAGGGCC
CP128824.1   AACGGGAATA TGTATGTCCG GGTGGCCAGT TGACTGGGTT GTAGCAGTGC TGCAGCTACG AATGTTAGAG ACAAAATGTA GTCCAGGGCC
CP025165.1   AACGGGAATA TGTATGTCCG GGTGGCCAGT TGACTGGGTT GTAGCAGTGC TGCAGCTACG AATGTTAGAG ACAAAATGTA GTCCAGGGCC
CP025157.1   AACGGGAATA TGTATGTCCG GGTGGCCAGT TGACTGGGTT GTAGCAGTGC TGCAGCTACG AATGTTAGAG ACAAAATGTA GTCCAGGGCC
CP025182.1   AACGGGAATA TGTATGTCCG GGTGGCCAGT TGACTGGGTT GTAGCAGTGC TGCAGCTACG AATGTTAGAG ACAAAATGTA GTCCAGGGCC
CP025174.1   AACGGGAATA TGTATGTCCG GGTGGCCAGT TGACTGGGTT GTAGCAGTGC TGCAGCTACG AATGTTAGAG ACAAAATGTA GTCCAGGGCC
CP017630.1   AACGGGAATA TGTATGTCCG GGTGGCCAGT TGACTGGGTT GTAGCAGTGC TGCAGCTACG AATGTTAGAG ACAAAATGTA GTCCAGGGCC
CP032012.1   AACGGGAATA TGTATGTTGG GGTGGCCAGT TGACTGGGTT GTAGCAGTGC TGCAGCTACG AATGTTAGAG ACAAAATGCA GTCCAGGACC
CP032012.1   AACGGGAATA TGTATGTTGG GGTGGCCAGT TGACTGGGTT GTAGCAGTGC TGCAGCTACG AATGTTAGAG ACAAAATGCA GTCCAGGACC
CaNTS2-F     ---------- ---------- ---------- ---------- ---------- ---------- ---------- ---------- ----------
CaNTS2-R     ---------- ---------- ---------- ---------- ---------- ---------- ---------- ---------- ----------

             ....|....| ....|....| ....|....| ....|....| ....|....| ....|....| ....|....| ....|....| ....|....|
                 275        285        295        305        315        325        335        345        355
FN554376.1   GGCCAGACGC AGTGTGCGTG CTGGTTGTGC AATTATACTA GCACATCTAG GTGTTTTAGG AAGAAACGTC CACCACCAAA AAATTAATTT
FN554375.1   GGCCAGACGC AGTGTGCGTG CTGGTTGTGC AATTATACTA GCACATCTAG GTGTTTTAGG AAGAAACGTC CACCACCAAA AAATTAATTT
CP128824.1   GGCCAGACGC AGTGTGCGTG CTGGTTGTGC AATTATACTA GCACATCTAG GTGTTTTAGG AAGAAACGTC CACCACCAAA AAATTAATTT
CP128824.1   GGCCAGACGC AGTGTGCGTG CTGGTTGTGC AATTATACTA GCACATCTAG GTGTTTTAGG AAGAAACGTC CACCACCAAA AAATTAATTT
CP128824.1   GGCCAGACGC AGTGTGCGTG CTGGTTGTGC AATTATACTA GCACATCTAG GTGTTTTAGG AAGAAACGTC CACCACCAAA AAATTAATTT
CP025165.1   GGCCAGACGC AGTGTGCGTG CTGGTTGTGC AATTATACTA GCACATCTAG GTGTTTTAGG AAGAAACGTC CACCACCAAA AAATTAATTT
CP025157.1   GGCCAGACGC AGTGTGCGTG CTGGTTGTGC AATTATACTA GCACATCTAG GTGTTTTAGG AAGAAACGTC CACCACCAAA AAATTAATTT
CP025182.1   GGCCAGACGC AGTGTGCGTG CTGGTTGTGC AATTATACTA GCACATCTAG GTGTTTTAGG AAGAAACGTC CACCACCAAA AAATTAATTT
CP025174.1   GGCCAGACGC AGTGTGCGTG CTGGTTGTGC AATTATACTA GCACATCTAG GTGTTTTAGG AAGAAACGTC CACCACCAAA AAATTAATTT
CP017630.1   GGCCAGACGC AGTGTGCGTG CTGGTTGTGC AATTATACTA GCACATCTAG GTGTTTTAGG AAGAAACGTC CACCACCAAA AAATTAATTT
CP032012.1   GGCCAGACGC AGTGTGCGTG CTGGTTGTGC AATTATACTA GCACATCTAG GTGTTTTAGG AAGAAACGTC CACCACCAAA AAATTAATTT
CP032012.1   GGCCAGACGC AGTGTGCGTG CTGGTTGTGC AATTATACTA GCACATCTAG GTGTTTTAGG AAGAAACGTC CACCACCAAA AAATTAATTT
CaNTS2-F     ---------- ---------- ---------- ---------- ---------- ---------- ---------- ---------- ----------
CaNTS2-R     ---------- ---------- ---------- ---------- ---------- ---------- ---------- ---------- ----------

             ....|....| ....|....| ....|....| ....|....| ....|....| ....|....| ....|....| ....|....| ....|....|
                 365        375        385        395        405        415        425        435        445
FN554376.1   CCAAATATTG GCACTTTTAT TACACCAGTG GTGTTACACA GCCCCC-CAA ACACTATCCC GGTTGTTAGA TTGAAGTTTG TTTAACAAAA
FN554375.1   CCAAATATTG GCACTTTTAT TACACCAGTG GTGTTACACA GCCCCC-CAA ACACTATCCC GGTTGTTAGA TTGAAGTTTG TTTAACAAAA
```

International Journal of *Molecular Sciences*

MDPI

```
CP128824.1    CCAAATATTG GCACTTTTAT TACACCAGTG GTGTTACACA GCCCCC-AAA ACACTATCCC GGTTGTTAGA TTGAAGTTTG TCTAACAAAA
CP128824.1    CCAAATATTG GCACTTTTAT TACACCAGTG GTGTTACACA GCCCCC-AAA ACACTATCCC GGTTGTTAGA TTGAAGTTTG TCTAACAAAA
CP128824.1    CCAAATATTG GCACTTTTAT TACACCAGTG GTGTTACACA GCCCCC-AAA ACACTATCCC GGTTGTTAGA TTGAAGTTTG TCTAACAAAA
CP025165.1    CCAAATATTG GCACTTTTAT TACACCAGTG GTGTTACACA GCCCCC-AAA ACACTATCCC GGTTGTTAGA TTGAAGTTTG TCTAACAAAA
CP025157.1    CCAAATATTG GCACTTTTAT TACACCAGTG GTGTTACACA GCCCCC-AAA ACACTATCCC GGTTGTTAGA TTGAAGTTTG TCTAACAAAA
CP025182.1    CCAAATATTG GCACTTTTAT TACACCAGTG GTGTTACACA GCCCCC-AAA ACACTATCCC GGTTGTTAGA TTGAAGTTTG TCTAACAAAA
CP025174.1    CCAAATATTG GCACTTTTAT TACACCAGTG GTGTTACACA GCCCCC-AAA ACACTATCCC GGTTGTTAGA TTGAAGTTTG TCTAACAAAA
CP017630.1    CCAAATATTG GCACTTTTAT TACACCAGTG GTGTTACACA GCCCCC-AAA ACACTATCCC GGTTGTTAGA TTGAAGTTTG TCTAACAAAA
CP032012.1    CCAAATATTG GCACTTTTAT TACACCAGTG GTGTTACACA GCCCCCCAAA ACACTATCCC GGTTGTTAGA TTGAAGTTTG TCTAACAAAA
CP032012.1    CCAAATATTG GCACTTTTAT TACACCAGTG GTGTTACACA GCCCCC-AAA ACACTATCCC GGTTGTTAGA TTGAAGTTTG TCTAACAAAA
CaNTS2-F      ---------- ---------- ---------- ---------- ---------- ---------- ---------- ---------- ----------
CaNTS2-R      ---------- ---------- ---------- ---------- ---------- ---------- ---------- ---------- ----------

              ....|....| ....|....| ....|....| ....|....| ....|....| ....|....| ....|....| ....|....| ....|....|
                  455        465        475        485        495        505        515        525        535
FN554376.1    A--TTGGAAG TTCTATTTTT TACTTTTTGT ACAAAATTTG GCAAGAAAAT TGGAGTAAAA TATTTTTATA TAAATTTCAA ACTAAAAATA
FN554375.1    A--TTGGAAG TTCTATTTTT TACTTTTTGT ACAAAATTTG GCAAGAAAAT TGGAGTAAAA TATTTTTATA TAAATTTCAA ACTAAAAATA
CP128824.1    A--TTGGAAG TTCTATTTTT TACTTTTTGT ACAAAATTTG GCAAGAAAAT TGAAGTAAAA TATTTTTATA TAAATTTCAA ACTAAAAATA
CP128824.1    A--TTGGAAG TTCTATTTTT TACTTTTTGT ACAAAATTTG GCAAGAAAAT TGAAGTAAAA TATTTTTATA TAAATTTCAA ACTAAAAATA
CP128824.1    A--TTGGAAG TTCTATTTTT TACTTTTTGT ACAAAATTTG GCAAGAAAAT TGAAGTAAAA TATTTTTATA TAAATTTCAA ACTAAAAATA
CP025165.1    A--TTGGAAG TTCTATTTTT TACTTTTTGT ACAAAATTTG GCAAGAAAAT TGAAGTAAAA TATTTTTATA TAAATTTCAA ACTAAAAATA
CP025157.1    A--TTGGAAG TTCTATTTTT TACTTTTTGT ACAAAATTTG GCAAGAAAAT TGAAGTAAAA TATTTTTATA TAAATTTCAA ACTAAAAATA
CP025182.1    A--TTGGAAG TTCTATTTTT TACTTTTTGT ACAAAATTTG GCAAGAAAAT TGAAGTAAAA TATTTTTATA TAAATTTCAA ACTAAAAATA
CP025174.1    A--TTGGAAG TTCTATTTTT TACTTTTTGT ACAAAATTTG GCAAGAAAAT TGAAGTAAAA TATTTTTATA TAAATTTCAA ACTAAAAATA
CP017630.1    A--TTGGAAG TTCTATTTTT TACTTTTTGT ACAAAATTTG GCAAGAAAAT TGAAGTAAAA TATTTTTATA TAAATTTCAA ACTAAAAATA
CP032012.1    AAATTGGAAG TTCTATTTTT TACTTTTTGT ACAAAATTTG GCAAGAAAAT TGGAGTAAAA TATTTTTATA TAAATTTCAA ACTAAAAATA
CP032012.1    A--TTGGAAG TTCTATTTTT TACTTTTTGT ACAAAATTTG GCAAGAAAAT TGGAGTAAAA TATTTTTATA TAAATTTCAA ACTAAAAATA
CaNTS2-F      ---------- ---------- ---------- ---------- ---------- ---------- ---------- ---------- ----------
CaNTS2-R      ---------- ---------- ---------- ---------- ---------- ---------- ---------- ---------- ----------

              ....|....| ....|....| ....|....| ....|....| ....|....| ....|....| ....|....| ....|....| ....|....|
                  545        555        565        575        585        595        605        615        625
FN554376.1    TCCAAAACAT AAAATAATAC ACAAAATGAG ATGCTCTATT TAGCCAAACC AACCATTACG GGTGTGTTGT TTGGTGTTGT CTGACCATGG
FN554375.1    TCCAAAACAT AAAATAATAC ACAAAATGAG ATGCTCTATT TAGCCAAACC AACCATTACG GGTGTGTTGT TTGGTGTTGT CTGACCATGG
CP128824.1    TCCAAAACAT AAAATAATAC ACAAAATGAG ATGCTCTATT TAGCCAAACC AACCATTACG GGTGTGTTGT TTGGTGTTGT CTGACCATGG
CP128824.1    TCCAAAACAT AAAATAATAC ACAAAATGAG ATGCTCTATT TAGCCAAACC AACCATTACG GGTGTGTTGT TTGGTGTTGT CTGACCATGG
CP128824.1    TCCAAAACAT AAAATAATAC ACAAAATGAG ATGCTCTATT TAGCCAAACC AACCATTACG GGTGTGTTGT TTGGTGTTGT CTGACCATGG
CP025165.1    TCCAAAACAT AAAATAATAC ACAAAATGAG ATGCTCTATT TAGCCAAACC AACCATTACG GGTGTGTTGT TTGGTGTTGT CTGACCATGG
CP025157.1    TCCAAAACAT AAAATAATAC ACAAAATGAG ATGCTCTATT TAGCCAAACC AACCATTACG GGTGTGTTGT TTGGTGTTGT CTGACCATGG
CP025182.1    TCCAAAACAT AAAATAATAC ACAAAATGAG ATGCTCTATT TAGCCAAACC AACCATTACG GGTGTGTTGT TTGGTGTTGT CTGACCATGG
CP025174.1    TCCAAAACAT AAAATAATAC ACAAAATGAG ATGCTCTATT TAGCCAAACC AACCATTACG GGTGTGTTGT TTGGTGTTGT CTGACCATGG
CP017630.1    TCCAAAACAT AAAATAATAC ACAAAATGAG ATGCTCTATT TAGCCAAACC AACCATTACG GGTGTGTTGT TTGGTGTTGT CTGACCATGG
```

International Journal of Molecular Sciences

MDPI

```
CP032012.1   TCCAAAACAT AAAATAATAC ACAAAATGAG ATGCTCTATT TAGCCAAACC AACCATTACG GGTGTGTTGT TTGGTGTTGT CTGACCATGG
CP032012.1   TCCAAAACAT AAAATAATAC ACAAAATGAG ATGCTCTATT TAGCCAAACC AACCATTACG GGTGTGTTGT TTGGTGTTGT CTGACCATGG
CaNTS2-F     ---------- ---------- ---------- ---------- TAGCCAAACC AACCATTACG GGTG------ ---------- ----------
CaNTS2-R     ---------- ---------- ---------- ---------- ---------- ---------- ---------- ---------- ----------

             ....|....| ....|....| ....|....| ....|....| ....|....| ....|....| ....|....| ....|....| ....|....|
                  635        645        655        665        675        685        695        705        715
FN554376.1   GTATACCATT TGTTAGTGTA TAGCTGCACT GTTTTATGTT TTTCGATTTT TCCACATAAA AGGAGTACTC CTATACTAAT AACAAACACT
FN554375.1   GTATACCATT TGTTAGTGTA TAGCTGCACT GTTTTATGTT TTTCGATTTT TCCACATAAA AGGAGTACTC CTATACTAAT AACAAACACT
CP128824.1   GTATACCATT TGTTAGTGTA TAGCTGCACT GTTTTATGTT TTTCGATTTT TCCACATAAA AGGAGTACTC CTATACTAAT AACAAACACT
CP128824.1   GTATACCATT TGTTAGTGTA TAGCTGCACT GTTTTATGTT TTTCGATTTT TCCACATAAA AGGAGTACTC CTATACTAAT AACAAACACT
CP128824.1   GTATACCATT TGTTAGTGTA TAGCTGCACT GTTTTATGTT TTTCGATTTT TCCACATAAA AGGAGTACTC CTATACTAAT AACAAACACT
CP025165.1   GTATACCATT TGTTAGTGTA TAGCTGCACT GTTTTATGTT TTTCGATTTT TCCACATAAA AGGAGTACTC CTATACTAAT AACAAACACT
CP025157.1   GTATACCATT TGTTAGTGTA TAGCTGCACT GTTTTATGTT TTTCGATTTT TCCACATAAA AGGAGTACTC CTATACTAAT AACAAACACT
CP025182.1   GTATACCATT TGTTAGTGTA TAGCTGCACT GTTTTATGTT TTTCGATTTT TCCACATAAA AGGAGTACTC CTATACTAAT AACAAACACT
CP025174.1   GTATACCATT TGTTAGTGTA TAGCTGCACT GTTTTATGTT TTTCGATTTT TCCACATAAA AGGAGTACTC CTATACTAAT AACAAACACT
CP017630.1   GTATACCATT TGTTAGTGTA TAGCTGCACT GTTTTATGTT TTTCGATTTT TCCACATAAA AGGAGTACTC CTATACTAAT AACAAACACT
CP032012.1   GTATACCATT TGTTAGTGTA TAGCTGCACT GTTTTATGTT TTTCGATTTT TCCACATAAA AGGAGTACTC CTATACTAAT AACAAACACT
CP032012.1   GTATACCATT TGTTAGTGTA TAGCTGCACT GTTTTATGTT TTTCGATTTT TCCACATAAA AGGAGTACTC CTATACTAAT AACAAACACT
CaNTS2-F     ---------- ---------- ---------- ---------- ---------- ---------- ---------- ---------- ----------
CaNTS2-R     ---------- ---------- ---------- ---------- ---------- ---------- ---------- ---------- ----------

             ....|....| ....|....| ....|....| ....|....| ....|....| ....|....| ....|....| ....|....| ....|....|
                  725        735        745        755        765        775        785        795        805
FN554376.1   CCACTATAAT TGGCAACCAC AATTATGCCT GGGGAAATAT TTTCCACTCC GAGTGTACAT ACATTTTTGG TGTGTTGTTG GTGGTTGAAA
FN554375.1   CCACTATAAT TGGCAACCAC AATTATGCCT GGGGAAATAT TTTCCACTCC GAGTGTACAT ACATTTTTGG TGTGTTGTTG GTGGTTGAAA
CP128824.1   CCACTATAAT TGGCAACCAC AATTATGCCT GGGGAAATAT TTTCCACTCC GAGTGTACAT ACATTTTTGG TGTGTTGTTG GTGGTTGAAA
CP128824.1   CCACTATAAT TGGCAACCAC AATTATGCCT GGGGAAATAT TTTCCACTCC GAGTGTACAT ACATTTTTGG TGTGTTGTTG GTGGTTGAAA
CP128824.1   CCACTATAAT TGGCAACCAC AATTATGCCT GGGGAAATAT TTTCCACTCC GAGTGTACAT ACATTTTTGG TGTGTTGTTG GTGGTTGAAA
CP025165.1   CCACTATAAT TGGCAACCAC AATTATGCCT GGGGAAATAT TTTCCACTCC GAGTGTACAT ACATTTTTGG TGTGTTGTTG GTGGTTGAAA
CP025157.1   CCACTATAAT TGGCAACCAC AATTATGCCT GGGGAAATAT TTTCCACTCC GAGTGTACAT ACATTTTTGG TGTGTTGTTG GTGGTTGAAA
CP025182.1   CCACTATAAT TGGCAACCAC AATTATGCCT GGGGAAATAT TTTCCACTCC GAGTGTACAT ACATTTTTGG TGTGTTGTTG GTGGTTGAAA
CP025174.1   CCACTATAAT TGGCAACCAC AATTATGCCT GGGGAAATAT TTTCCACTCC GAGTGTACAT ACATTTTTGG TGTGTTGTTG GTGGTTGAAA
CP017630.1   CCACTATAAT TGGCAACCAC AATTATGCCT GGGGAAATAT TTTCCACTCC GAGTGTACAT ACATTTTTGG TGTGTTGTTG GTGGTTGAAA
CP032012.1   CCACTATAAT TGGCAACCAC AATTATGCCT GGGGAAATAT TTTCCACTCC GAGTGTACAT ACATTTTTGG TGTGTTGTTG GTGGTTGAAA
CP032012.1   CCACTATAAT TGGCAACCAC AATTATGCCT GGGGAAATAT TTTCCACTCC GAGTGTACAT ACATTTTTGG TGTGTTGTTG GTGGTTGAAA
CaNTS2-F     ---------- ---------- ---------- ---------- ---------- ---------- ---------- ---------- ----------
CaNTS2-R     ---------- ---------- ---------- ---------- ---------- ---------- ---------- ---------- ----------

             ....|....| ....|....| ....|....| ....|....| ....|....| ....|....| ....|....| ....|....| ....|....|
                  815        825        835        845        855        865        875        885        895
FN554376.1   ATTGTGTGGG TCACGTGCAT TTAATTAGGG TTAGGGCTAC GGTTTGTGTG CAGTACCACG TTCAAATTGA GGCTCTCTGG TATAACTTGC
```

International Journal of *Molecular Sciences*

MDPI

```
FN554375.1    ATTGTGTGGG TCACGTGCAT TTAATTAGGG TTAGGGCTAC GGTTTGTGTG CAGTACCACG TTCAAATTGA GGCTCTCTGG TATAACTTGC
CP128824.1    ATTGTGTGGG TCACGTGCAT TTAATTAGGG TTAGGGCTAC GGTTTGTGTG CAGTACCACG TTCAAATTGA GGCTCTCTGG TATAACTTGC
CP128824.1    ATTGTGTGGG TCACGTGCAT TTAATTAGGG TTAGGGCTAC GGTTTGTGTG CAGTACCACG TTCAAATTGA GGCTCTCTGG TATAACTTGC
CP128824.1    ATTGTGTGGG TCACGTGCAT TTAATTAGGG TTAGGGCTAC GGTTTGTGTG CAGTACCACG TTCAAATTGA GGCTCTCTGG TATAACTTGC
CP025165.1    ATTGTGTGGG TCACGTGCAT TTAATTAGGG TTAGGGCTAC GGTTTGTGTG CAGTACCACG TTCAAATTGA GGCTCTCTGG TATAACTTGC
CP025157.1    ATTGTGTGGG TCACGTGCAT TTAATTAGGG TTAGGGCTAC GGTTTGTGTG CAGTACCACG TTCAAATTGA GGCTCTCTGG TATAACTTGC
CP025182.1    ATTGTGTGGG TCACGTGCAT TTAATTAGGG TTAGGGCTAC GGTTTGTGTG CAGTACCACG TTCAAATTGA GGCTCTCTGG TATAACTTGC
CP025174.1    ATTGTGTGGG TCACGTGCAT TTAATTAGGG TTAGGGCTAC GGTTTGTGTG CAGTACCACG TTCAAATTGA GGCTCTCTGG TATAACTTGC
CP017630.1    ATTGTGTGGG TCACGTGCAT TTAATTAGGG TTAGGGCTAC GGTTTGTGTG CAGTACCACG TTCAAATTGA GGCTCTCTGG TATAACTTGC
CP032012.1    ATTGTGTGGG TCACGTGCAT TTAATTAGGG TTAGGGCTAC GGTTTGTGTG CAGTACCACG TTCAAATTGA GGCTCTCTGG TATAACTTGC
CP032012.1    ATTGTGTGGG TCACGTGCAT TTAATTAGGG TTAGGGCTAC GGTTTGTGTG CAGTACCACG TTCAAATTGA GGCTCTCTGG TATAACTTGC
CaNTS2-F      ---------- ---------- ---------- ---------- ---------- ---------- ---------- ---------- ----------
CaNTS2-R      ---------- ---------- ---------- ---------- ---------G CAGTACCACG TTCAAATTGA GGC------- ----------
```

(b) Diagrammatic alignment representation of CpNTS2 forward and reverse primers and *Candida parapsilosis* NTS2 region (partial) of rDNA locus. Computed by Multiple Sequence Alignment ClustalW algorithim.

```
              ....|....| ....|....| ....|....| ....|....| ....|....| ....|....| ....|....| ....|....| ....|....|
                   5         15         25         35         45         55         65         75         85
FN554241.1    GAAGTACTCT CATTTTGAGG TGCCTATGGT GGGTAGTGAC AGGTAACGTG ACCTCATTTT GCGGGAGTGA AATTTTTCTT TTTTTACCAG
LT596114.1    GAAATACTCT CATTTTGAGG TGCCTATGGT GGGTAGTGAC AGGTAACGTG ACCTCATTTT GCGGGAGTGA AATTTTTCTT TTTTTACCAG
LT596101.1    GAAATACTCT CATTTTGAGG TGCCTATGGT GGGTAGTGAC AGGTAACGTG ACCTCATTTT GCGGGAGTGA AATTTTTCTT TTTTTACCAG
LT596100.1    GAAATACTCT CATTTTGAGG TGCCTATGGT GGGTAGTGAC AGGTAACGTG ACCTCATTTT GCGGGAGTGA AATTTTTCTT TTTTTACCAG
LT596099.1    GAAATACTCT CATTTTGAGG TGCCTATGGT GGGTAGTGAC AGGTAACGTG ACCTCATTTT GCGGGAGTGA AATTTTTCTT TTTTTACCAG
LT596098.1    GAAATACTCT CATTTTGAGG TGCCTATGGT GGGTAGTGAC AGGTAACGTG ACCTCATTTT GCGGGAGTGA AATTTTTCTT TTTTTACCAG
LT596097.1    GAAATACTCT CATTTTGAGG TGCCTATGGT GGGTAGTGAC AGGTAACGTG ACCTCATTTT GCGGGAGTGA AATTTTTCTT TTTTTACCAG
LT596096.1    GAAATACTCT CATTTTGAGG TGCCTATGGT GGGTAGTGAC AGGTAACGTG ACCTCATTTT GCGGGAGTGA AATTTTTCTT TTTTTACCAG
LT596095.1    GAAATACTCT CATTTTGAGG TGCCTATGGT GGGTAGTGAC AGGTAACGTG ACCTCATTTT GCGGGAGTGA AATTTTTCTT TTTTTACCAG
LT596094.1    GAAATACTCT CATTTTGAGG TGCCTATGGT GGGTAGTGAC AGGTAACGTG ACCTCATTTT GCGGGAGTGA AATTTTTCTT TTTTTACCAG
HE605209.1    GAAATACTCT CATTTTGAGG TGCCTATGGT GGGTAGTGAC AGGTAACGTG ACCTCATTTT GCGGGAGTGA AATTTTTCTT TTTTTACCAG
FN554242.1    GAAATACTCT CATTTTGAGG TGCCTATGGT GGGTAGTGAC AGGTAACGTG ACCTCATTTT GCGGGAGTGA AATTTTTCTT TTTTTACCAG
CpNTS2-F      ---------- ---------- ---------- ---------- ---------- ---------- ---------- ---------- ----------
CpNTS2-R      ---------- ---------- ---------- ---------- ---------- ---------- ---------- ---------- ----------

              ....|....| ....|....| ....|....| ....|....| ....|....| ....|....| ....|....| ....|....| ....|....|
                    95        105        115        125        135        145        155        165        175
FN554241.1    AGCATAACAC CACATATCAG AAGATCAAAG GCAACAAAAA TTTTCCTTTT CTTTTTACTC TTCTTTCTTT TATGTTAGGC GAGACTTGCA
LT596114.1    AGCATAACAC CACATATCAG AAGATCAAAG GCAACAAAAA TTTTCCTTTT CTTTTTACTC TTCTTTCTTT TATGTTAGGC GAGACTTGCA
LT596101.1    AGCATAACAC CACATATCAG AAGATCAAAG GCAACAAAAA TTTTCCTTTT CTTTTTACTC TTCTTTCTTT TATGTTAGGC GAGACTTGCA
LT596100.1    AGCATAACAC CACATATCAG AAGATCAAAG GCAACAAAAA TTTTCCTTTT CTTTTTACTC TTCTTTCTTT TATGTTAGGC GAGACTTGCA
```

International Journal of Molecular Sciences

MDPI

```
LT596099.1   AGCATAACAC CACATATCAG AAGATCAAAG GCAACAAAAA TTTTCCTTTT CTTTTTACTC TTCTTTCTTT TATGTTAGGC GAGACTTGCA
LT596098.1   AGCATAACAC CACATATCAG AAGATCAAAG GCAACAAAAA TTTTCCTTTT CTTTTTACTC TTCTTTCTTT TATGTTAGGC GAGACTTGCA
LT596097.1   AGCATAACAC CACATATCAG AAGATCAAAG GCAACAAAAA TTTTCCTTTT CTTTTTACTC TTCTTTCTTT TATGTTAGGC GAGACTTGCA
LT596096.1   AGCATAACAC CACATATCAG AAGATCAAAG GCAACAAAAA TTTTCCTTTT CTTTTTACTC TTCTTTCTTT TATGTTAGGC GAGACTTGCA
LT596095.1   AGCATAACAC CACATATCAG AAGATCAAAG GCAACAAAAA TTTTCCTTTT CTTTTTACTC TTCTTTCTTT TATGTTAGGC GAGACTTGCA
LT596094.1   AGCATAACAC CACATATCAG AAGATCAAAG GCAACAAAAA TTTTCCTTTT CTTTTTACTC TTCTTTCTTT TATGTTAGGC GAGACTTGCA
HE605209.1   AGCATAACAC CACATATCAG AAGATCAAAG GCAACAAAAA TTTTCCTTTT CTTTTTACTC TTCTTTCTTT TATGTTAGGC GAGACTTGCA
FN554242.1   AGCATAACAC CACATATCAG AAGATCAAAG GCAACAAAAA TTTTCCTTTT CTTTTTACTC TTCTTTCTTT TATGTTAGGC GAGACTTGCA
CpNTS2-F     ---------- ---------- ---------- ---------- ---------- ---------- ---------- ---------- ----------
CpNTS2-R     ---------- ---------- ---------- ---------- ---------- ---------- ---------- ---------- ----------

             ....|....| ....|....| ....|....| ....|....| ....|....| ....|....| ....|....| ....|....| ....|....|
                  185        195        205        215        225        235        245        255        265
FN554241.1   CAAGATTATG ATATGTGCGA CCACTCTCCG CGCTAGTTCT TCCCTGATGC CACCAACACC CATGTTAACA TCATCATCAA CAACACACAA
LT596114.1   CAAGATTATG ATATGTGCGA CCACTCTCCG CGCTAGTTCT TCCCTGATGC CACCAACACC CATGTTAACA TCATCATCAA CAACACACAA
LT596101.1   CAAGATTATG ATATGTGCGA CCACTCTCCG CGCTAGTTCT TCCCTGATGC CACCAACACC CATGTTAACA TCATCATCAA CAACACACAA
LT596100.1   CAAGATTATG ATATGTGCGA CCACTCTCCG CGCTAGTTCT TCCCTGATGC CACCAACACC CATGTTAACA TCATCATCAA CAACACACAA
LT596099.1   CAAGATTATG ATATGTGCGA CCACTCTCCG CGCTAGTTCT TCCCTGATGC CACCAACACC CATGTTAACA TCATCATCAA CAACACACAA
LT596098.1   CAAGATTATG ATATGTGCGA CCACTCTCCG CGCTAGTTCT TCCCTGATGC CACCAACACC CATGTTAACA TCATCATCAA CAACACACAA
LT596097.1   CAAGATTATG ATATGTGCGA CCACTCTCCG CGCTAGTTCT TCCCTGATGC CACCAACACC CATGTTAACA TCATCATCAA CAACACACAA
LT596096.1   CAAGATTATG ATATGTGCGA CCACTCTCCG CGCTAGTTCT TCCCTGATGC CACCAACACC CATGTTAACA TCATCATCAA CAACACACAA
LT596095.1   CAAGATTATG ATATGTGCGA CCACTCTCCG CGCTAGTTCT TCCCTGATGC CACCAACACC CATGTTAACA TCATCATCAA CAACACACAA
LT596094.1   CAAGATTATG ATATGTGCGA CCACTCTCCG CGCTAGTTCT TCCCTGATGC CACCAACACC CATGTTAACA TCATCATCAA CAACACACAA
HE605209.1   CAAGATTATG ATATGTGCGA CCACTCTCCG CGCTAGTTCT TCCCTGATGC CACCAACACC CATGTTAACA TCATCATCAA CAACACACAA
FN554242.1   CAAGATTATG ATATGTGCGA CCACTCTCCG CGCTAGTTCT TCCCTGATGC CACCAACACC CATGTTAACA TCATCATCAA CAACACACAA
CpNTS2-F     ---------- ---------- ---------- ---------- -CCCTGATGC CACCAACACC ---------- ---------- ----------
CpNTS2-R     ---------- ---------- ---------- ---------- ---------- ---------- ---------- ---------- ----------

             ....|....| ....|....| ....|....| ....|....| ....|....| ....|....| ....|....| ....|....| ....|....|
                  275        285        295        305        315        325        335        345        355
FN554241.1   ACAACATTTT CACCAAAAGA GCAAGCGTTC GTCACGACAA CCTACCTCCC CAAATACATC TATGATGCTT TTAAGTTTGT AAGTTTTGTT
LT596114.1   ACAACATTTT CACCAAAAGA GCAAGCGTTC GTCACGACAA CCTACCTCCC CAAATACATC TATGATGCTT TTAAGTTTGT AAGTTTTGTT
LT596101.1   ACAACATTTT CACCAAAAGA GCAAGCGTTC GTCACGACAA CCTACCTCCC CAAATACATC TATGATGCTT TTAAGTTTGT AAGTTTTGTT
LT596100.1   ACAACATTTT CACCAAAAGA GCAAGCGTTC GTCACGACAA CCTACCTCCC CAAATACATC TATGATGCTT TTAAGTTTGT AAGTTTTGTT
LT596099.1   ACAACATTTT CACCAAAAGA GCAAGCGTTC GTCACGACAA CCTACCTCCC CAAATACATC TATGATGCTT TTAAGTTTGT AAGTTTTGTT
LT596098.1   ACAACATTTT CACCAAAAGA GCAAGCGTTC GTCACGACAA CCTACCTCCC CAAATACATC TATGATGCTT TTAAGTTTGT AAGTTTTGTT
LT596097.1   ACAACATTTT CACCAAAAGA GCAAGCGTTC GTCACGACAA CCTACCTCCC CAAATACATC TATGATGCTT TTAAGTTTGT AAGTTTTGTT
LT596096.1   ACAACATTTT CACCAAAAGA GCAAGCGTTC GTCACGACAA CCTACCTCCC CAAATACATC TATGATGCTT TTAAGTTTGT AAGTTTTGTT
LT596095.1   ACAACATTTT CACCAAAAGA GCAAGCGTTC GTCACGACAA CCTACCTCCC CAAATACATC TATGATGCTT TTAAGTTTGT AAGTTTTGTT
LT596094.1   ACAACATTTT CACCAAAAGA GCAAGCGTTC GTCACGACAA CCTACCTCCC CAAATACATC TATGATGCTT TTAAGTTTGT AAGTTTTGTT
HE605209.1   ACAACATTTT CACCAAAAGA GCAAGCGTTC GTCACGACAA CCTACCTCCC CAAATACATC TATGATGCTT TTAAGTTTGT AAGTTTTGTT
FN554242.1   ACAACATTTT CACCAAAAGA GCAAGCGTTC GTCACGACAA CCTACCTCCC CAAATACATC TATGATGCTT TTAAGTTTGT AAGTTTTGTT
```

```
CpNTS2-F      ---------- ---------- ---------- ---------- ---------- ---------- ---------- ---------- ----------
CpNTS2-R      ---------- ---------- ---------- ---------- ---------- ---------- ---------- ---------- ----------

              ....|....| ....|....| ....|....| ....|....| ....|....| ....|....| ....|....| ....|....| ....|....|
                   365        375        385        395        405        415        425        435        445
FN554241.1    TTCTTTGTCT CGACCTATTT TCGACCTAAA TCGGTTGACC CTCTACGTCG ATATTTATCT TCTTACAACG ACGCTCTAGC ACGGTGTCTG
LT596114.1    TTCTTTGTCT CGACCTATTT TCGACCTAAA TCGGTTGACC CTCTACGTCG ATATTTATCT TCTTACAACG ACGCTCTAGC ACGGTGTCTG
LT596101.1    TTCTTTGTCT CGACCTATTT TCGACCTAAA TCGGTTGACC CTCTACGTCG ATATTTATCT TCTTACAACG ACGCTCTAGC ACGGTGTCTG
LT596100.1    TTCTTTGTCT CGACCTATTT TCGACCTAAA TCGGTTGACC CTCTACGTCG ATATTTATCT TCTTACAACG ACGCTCTAGC ACGGTGTCTG
LT596099.1    TTCTTTGTCT CGACCTATTT TCGACCTAAA TCGGTTGACC CTCTACGTCG ATATTTATCT TCTTACAACG ACGCTCTAGC ACGGTGTCTG
LT596098.1    TTCTTTGTCT CGACCTATTT TCGACCTAAA TCGGTTGACC CTCTACGTCG ATATTTATCT TCTTACAACG ACGCTCTAGC ACGGTGTCTG
LT596097.1    TTCTTTGTCT CGACCTATTT TCGACCTAAA TCGGTTGACC CTCTACGTCG ATATTTATCT TCTTACAACG ACGCTCTAGC ACGGTGTCTG
LT596096.1    TTCTTTGTCT CGACCTATTT TCGACCTAAA TCGGTTGACC CTCTACGTCG ATATTTATCT TCTTACAACG ACGCTCTAGC ACGGTGTCTG
LT596095.1    TTCTTTGTCT CGACCTATTT TCGACCTAAA TCGGTTGACC CTCTACGTCG ATATTTATCT TCTTACAACG ACGCTCTAGC ACGGTGTCTG
LT596094.1    TTCTTTGTCT CGACCTATTT TCGACCTAAA TCGGTTGACC CTCTACGTCG ATATTTATCT TCTTACAACG ACGCTCTAGC ACGGTGTCTG
HE605209.1    TTCTTTGTCT CGACCTATTT TCGACCTAAA TCGGTTGACC CTCTACGTCG ATATTTATCT TCTTACAACG ACGCTCTAGC ACGGTGTCTG
FN554242.1    TTCTTTGTCT CGACCTATTT TCGACCTAAA TCGGTTGACC CTCTACGTCG ATATTTATCT TCTTACAACG ACGCTCTAGC ACGGTGTCTG
CpNTS2-F      ---------- ---------- ---------- ---------- ---------- ---------- ---------- ---------- ----------
CpNTS2-R      ---------- ---------- ---------- ---------- ---------- ---------T TCTTACAACG ACGCTCTAGC ----------
```

(c) Diagrammatic alignment representation of CtNTS2 forward and reverse primers and *Candida tropicalis* NTS2 region (partial) of rDNA locus. Computed by Multiple Sequence Alignment ClustalW algorithim.

```
              ....|....| ....|....| ....|....| ....|....| ....|....| ....|....| ....|....| ....|....| ....|....|
                   5          15         25         35         45         55         65         75         85
FN554382.1    GAAAAATTTC TGATTTTGGT TTTTATATAT GCTGAGACAC GACTTTTTCG CATTTTTCTC AAAACACCAT AAAGTTGTTA GCAACAGTGG
FN554383.1    GAAAAATTTC TGATTTTGGT TTTTATATAT GCTGAGACAC GACTTTTTCG CATTTTTCTC AAAACACCAT AAAGTTGTTA GCAACAGTGG
KF569951.1    --AAAATTTC TGATTTTGGT TTTTATATAT GCTGAGACAC GACTTTTTCG CATTTTTCTC AAACCACCAT AAAGTTGTTA GCAACAGTGG
CtNTS2-F      ---------- ---------- ---------- ---------- ---------- ---------- ---------- ---------- ----------
CtNTS2-R      ---------- ---------- ---------- ---------- ---------- ---------- ---------- ---------- ----------

              ....|....| ....|....| ....|....| ....|....| ....|....| ....|....| ....|....| ....|....| ....|....|
                   95         105        115        125        135        145        155        165        175
FN554382.1    TGGTTTTTCA AGTGGTATAG GGTAATTTGG GGCGTAGAAT TCGATGGGAG TGATAAAAGT AGGAAGTTTC GGGGTATATG TTGGTGAAAT
FN554383.1    TGGTTTTGCA AGTGGTATAG GGTAATTTGG GGCGTAGAAT TCGATGGGAG TGATAAAAGT AGGAAGTTTC GGGGTATATG TTGGTGAAAT
KF569951.1    TGGTTTTGCA AGTGGTATAG GGTAATTTGG GGCGTAGAAT TCGATGGGAG TGATAAAAGT AGGAAGTTTC GGGGTATATG TTGGTGAAAT
CtNTS2-F      ---------- ---------- ---------G GGCGTAGAAT TCGATGGGAG TGA------- ---------- ---------- ----------
CtNTS2-R      ---------- ---------- ---------- ---------- ---------- ---------- ---------- ---------- ----------

              ....|....| ....|....| ....|....| ....|....| ....|....| ....|....| ....|....| ....|....| ....|....|
```

International Journal of
Molecular Sciences

MDPI

```
               185        195        205        215        225        235        245        255        265
FN554382.1    GGCCGTGTCA CGTGACGCAC TCTTCAGATG ACCGACTTGC CGCAGTGCGA TTGGTCAATT ATGGAATGGA GATGGTGTAA CCAGGCCCGG
FN554383.1    GGCCATGTCA CGTGACGCAC TCTTCAGATT ACCGACTTGC CGCAGTGCGA TTGGTCAATT ATGGAATGGA GATGGTGTAA CCAGACCCGG
KF569951.1    GGCCGTGTCA CGTGATGAAC TCTTCAGATT ACCGACTTGC CGCAGTGCAA TCAATCAATT ATGGAATGGA GATGGTGTAA CCAGACCCGG
CtNTS2-F      ---------- ---------- ---------- ---------- ---------- ---------- ---------- ---------- ----------
CtNTS2-R      ---------- ---------- ---------- ---------- ---------- ---------- ---------- ---------- ----------

              ....|....| ....|....| ....|....| ....|....| ....|....| ....|....| ....|....| ....|....| ....|....|
               275        285        295        305        315        325        335        345        355
FN554382.1    CGAGTAAAAT GAGACCTTAT GTGTGGTCAT AGTGGTTATA GTTGGTGAGT TATTGGTATA TATGTGTTGG GTGAAAATTT CAAACTGCGT
FN554383.1    CGAGTAAAAT GAGACCTTAT GTGTGGTCAT AGTGGTTATA GTTGGTGAGT TATCTGGTCA TATGTGTTGG GCGAAAATTT CAAAATGCGT
KF569951.1    CGAGTAAAAT GAGACCTTAT GTGTGGTCAT AGTGGTTATA GTTGGTGAGT TATCTGGACA TATGTGTTGG GCGAAAATTT CAAAATGCGT
CtNTS2-F      ---------- ---------- ---------- ---------- ---------- ---------- ---------- ---------- ----------
CtNTS2-R      ---------- ---------- ---------- ---------- ---------- ---------- ---------- ---------- ----------

              ....|....| ....|....| ....|....| ....|....| ....|....| ....|....| ....|....| ....|....| ....|....|
               365        375        385        395        405        415        425        435        445
FN554382.1    TTAAGATAGT GGTTATAATT TTCATAGTAA CTAGACACTC TAGTAAGCCC CTCCCAAGTG TCAAAGTTTG ACTTTTTGCA TTGGTTGAAG
FN554383.1    TTAAGATAGT GGTTATAATT TTCATAGTAA CTAGACACTC TAGTAAGCCC CTCCCAAGTG TCAAAGTTTG ACTTTTTGCA TTGGTTGAAG
KF569951.1    TTAAGATAGT GGTTATAATT TTCATAGTAA CTAGACACTC TAGTAAGCCC CTCCCAAGTG TCAAAGTTTG ACTTTTTGCA TTGGTTGAAG
CtNTS2-F      ---------- ---------- ---------- ---------- ---------- ---------- ---------- ---------- ----------
CtNTS2-R      ---------- ---------- ---------- --------TC TAGTAAGCCC CTCCCAAGTG TC-------- ---------- ----------
```

(d) Diagrammatic alignment representation of NgNTS2 forward and reverse primers and *Nakazeomyces glabratus* NTS2 region (partial) of rDNA locus. Computed by Multiple Sequence Alignment ClustalW algorithim.

```
              ....|....| ....|....| ....|....| ....|....| ....|....| ....|....| ....|....| ....|....| ....|....|
                  5         15         25         35         45         55         65         75         85
LT837783.1    GAAGTACCCC CGGACCGAGC TTGCTCTTCG GCCTGGCTCA AAAAGTAGCC AAAGGGCCCG GCATCTGGCA TCCGCAATCC ACTTTGAACA
CP048130.1    GAAGTACCCC CGGACCGAGC TTGCTCTTCG GCCTGGCTCG AAAAGTAGCC AAAGGGCCCG GCATCTGGCA TCTGCAGTCC ACTTTGAACA
CP048130.1    GAAGTACCCC CGGACCGAGC TTGCTCTTCG GCCTGGCTCG AAAAGTAGCC AAAGGGCCCG GCATCTGGCA TCTGCAGTCC ACTTTGAACA
CP048130.1    GAAGTACCCC CGGACCGAGC TTGCTCTTCG GCCTGGCTCG AAAAGTAGCC AAAGGGCCCG GCATCTGGCA TCTGCAGTCC ACTTTGAACA
CP048129.1    GAAGTACCCC CGGACCGAGC TTGCTCTTCG GCCTGGCTCG AAAAGTAGCC AAAGGGCCCG GCATCTGGCA TCTGCAGTCC ACTTTGAACA
CP048129.1    GAAGTACCCC CGGACCGAGC TTGCTCTTCG GCCTGGCTCG AAAAGTAGCC AAAGGGCCCG GCATCTGGCA TCTGCAGTCC ACTTTGAACA
CP048129.1    GAAGTACCCC CGGACCGAGC TTGCTCTTCG GCCTGGCTCG AAAAGTAGCC AAAGGGCCCG GCATCTGGCA TCTGCAGTCC ACTTTGAACA
CP089623.1    GAAGTACCCC CGGACCGAGC TTGCTCTTCG GCCTGGCTCG AAAAGTAGCC AAAGGGCCCG GCATCTGGCA TCTGCAGTCC ACTTTGAACA
CP089623.1    GAAGTACCCC CGGACCGAGC TTGCTCTTCG GCCTGGCTCG AAAAGTAGCC AAAGGGCCCG GCATCTGGCA TCTGCAGTCC ACTTTGAACA
LT837773.1    GAAGTACCCC CGGACCGAGC TTGCTCTTCG GCCTGGCTCA AAAAGTAGCC AAAGGGCCCG GCATCTGGCA TCCGCAATCC ACTTTGAACA
LT837755.1    GAAGTACCCC CGGACCGAGC TTGCTCTTCG GCCTGGCTCG AAAAGTAGCC AAAGGGCCCG GCATCTGGCA TCTGCAATCC ACTTTGAACA
```

```
CR380958.2   GAAGTACCCC CGGACCGAGC TTGCTCTTCG GCCTGGCTCG AAAAGTAGCC AAAGGGCCCG GCATCTGGCA TCTGCAGTCC ACTTTGAACA
LT837789.1   GAAGTACCCC CGGATCGAGC TTGCTCTTCG GCCTGGCTCG AAAAGTAGCC AAAGGGCCCG GCATCTGGCA TCTGCAGTCC ACTTTGAACA
LT837788.1   GAAGTACCCC CGGACCGAGC TTGCTCTTCG GCCTGGCTCG AAAAGTAGCC AAAGGGCCCG GCATCTGGCA TCTGCAGTCC ACTTTGAACA
LT837787.1   GAAGTACCCC CGGACCGAGC TTGCTCTTCG GCCTGGCTCG AAAAGTAGCC AAAGGGCCCG GCATCTGGCA TCTGCAGTCC ACTTTGAACA
LT837781.1   GAAGTACCCC CGGACCGAGC TTGCTCTTCG GCCTGGCTCG AAAAGTAGCC AAAGGGCCCG GCATCTGGCA TCTGCAATCC ACTTTGAACA
LT837776.1   GAAGTACCCC CGGACCGAGC TTGCTCTTCG GCCTGGCTCG AAAAGTAGCC AAAGGGCCCG GCATCTGGCA TCTGCAATCC ACTTTGAACA
LT837782.1   GAAGTACCCC CGGACCGAGC TTGCTCTTCG GCCTGGCTTG AAAAGTAGCC AAAGGGCCCG GCATCTGGCA TCTGCAATCC ACTTTGAACA
LT837780.1   GAAGTACCCC CGGACCGAGC TTGCTCTTCG GCCTGGCTCG AAAAGTAGCC AAAGGGCCCG GCATCTGGCA TCTGCAATCC ACTTTGAACA
LT837775.1   GAAGTACCCC CGGACCGAGC TTGCTCTTCG GCCTGGCTCG AAAAGTAGCC AAAGGGCCCG GCATCTGGCA TCTGCAATCC ACTTTGAACA
LT837753.1   GAAGTACCCC CGGACCGAGC TTGCTCTTCG GCCTGGCTCG AAAAGTAGCC AAAGGGCCCG GCATCTGGCA TCTGCAATCC ACTTTGAACA
FN554379.1   GAAGTACCCC CGGACCGAGC TTGCTCTTCG GCCTGGCTCG AAAAGTAGCC AAAGGGCCCG GCATCTGGCA TCTGCAGTCC ACTTTGAACA
LT837754.1   GAAGTACCCC CGGACCGAGC TTGCTCTTCG GCCTGGCTCG AAAAGTAGCC AAAGGGCCCG GCATCTGGCA TCTGCAATCC ACTTTGAACA
CP089636.1   GAAGTACCCC CGGACCGAGC TTGCTCTTCG GCCTGGCTCA AAAAGTAGCC AAAGGGCCCG GCATCTGGCA TCCGCAATCC ACTTTGAACA
CP089636.1   GAAGTACCCC CGGACCGAGC TTGCTCTTCG GCCTGGCTCA AAAAGTAGCC AAAGGGCCCG GCATCTGGCA TCCGCAATCC ACTTTGAACA
CP089663.1   GAAGTACCCC CGGACCGAGC TTGCTCTTCG GCCTGGCTCG AAAAGTAGCC AAAGGGCCCG GCATCTGGCA TCTGCAGTCC ACTTTGAACA
LT837772.1   GAAGTACCCC CGGACCGAGC TTGCTCTTCG GCCTGGCTCG AAAAGTAGCC AAAGGGCCCG GCATCTGGCA TCTGCAATCC ACTTTGAACA
LT837757.1   GAAGTACCCC CGGACCGAGC TTGCTCTTCG GCCTGGCTCG AAAAGTAGCC AAAGGGCCCG GCATCTGGCA TCTGCAATCC ACTTTGAACA
LT837762.1   GAAGTACCCC CGGACCGAGC TTGCTCTTCG GCCTGGCTCG AAAAGTAGCC AAAGGGCCCG GCATCTGGCA TCTGCAATCC ACTTTGAACA
LT837761.1   GAAGTACCCC CGGACCGAGC TTGCTCTTCG GCCTGGCTCG AAAAGTAGCC AAAGGGCCCG GCATCTGGCA TCTGCAATCC ACTTTGAACA
CP089637.1   GAAGTACCCC CGGACCGAGC TTGCTCTTCG GCCTGGCTCA AAAAGTAGCC AAAGGGCCCG GCATCTGGCA TCCGCAATCC ACTTTGAACA
CP089637.1   GAAGTACCCC CGGACCGAGC TTGCTCTTCG GCCTGGCTCA AAAAGTAGCC AAAGGGCCCG GCATCTGGCA TCCGCAATCC ACTTTGAACA
LT837758.1   GAAGTACCCC CGGACCGAGC TTGCTCTTCG GCCTGGCTCG AAAAGTAGCC AAAGGGCCCG GCATCTGGCA TCTGCAATCC ACTTTGAACA
CP089715.1   GAAGTACCCC CGGACCGAGC TTGCTCTTCG GCCTGGCTCG AAAAGTAGCC AAAGGGCCCG GCATCTGGCA TCTGCAATCC ACTTTGAACA
CP089715.1   GAAGTACCCC CGGACCGAGC TTGCTCTTCG GCCTGGCTCG AAAAGTAGCC AAAGGGCCCG GCATCTGGCA TCTGCAATCC ACTTTGAACA
CP089715.1   GAAGTACCCC CGGACCGAGC TTGCTCTTCG GCCTGGCTCG AAAAGTAGCC AAAGGGCCCG GCATCTGGCA TCTGCAATCC ACTTTGAACA
LT837767.1   GAAGTACCCC CGGACCGAGC TTGCTCTTCG GCCTGGCTCG AAAAGTAGCC AAAGGGCCCG GCATCTGGCA TCTGCAATCC ACTTTGAACA
LT837765.1   GAAGTACCCC CGGACCGAGC TTGCTCTTCG GCCTGGCTCG AAAAGTAGCC AAAGGGCCCG GCATCTGGCA TCTGCAATCC ACTTTGAACA
CP089662.1   GAAGTACCCC CGGACCGAGC TTGCTCTTCG GCCTGGCTCG AAAAGTAGCC AAAGGGCCCG GCATCTGGCA TCTGCAGTCC ACTTTGAACA
CP089662.1   GAAGTACCCC CGGACCGAGC TTGCTCTTCG GCCTGGCTCG AAAAGTAGCC AAAGGGCCCG GCATCTGGCA TCTGCAGTCC ACTTTGAACA
LT837790.1   GAAGTACCCC CGGACCGAGC TTGCTCTTCG GCCTGGCTCG AAAAGTAGCC AAAGGGCCCG GCATCTGGCA TCTGCAGTCC ACTTTGAACA
LT837774.1   GAAGTACCCC CGGACCGAGC TTGCTCTTCG GCCTGGCTCG AAAAGTAGCC AAAGGGCCCG GCATCTGGCA TCTGCAATCC ACTTTGAACA
LT837751.1   GAAGTACCCC CGGACCGAGC TTGCTCTTCG GCCTGGCTCG AAAAGTAGCC AAAGGGCCCG GCATCTGGCA TCTGCAATCC ACTTTGAACA
CP048242.1   GAAGTACCCC CGGACCGAGC TTGCTCTTCG GCCTGGCTCG AAAAGTAGCC AAAGGGCCCG GCATCTGGCA TCTGCAGTCC ACTTTGAACA
CP048242.1   GAAGTACCCC CGGACCGAGC TTGCTCTTCG GCCTGGCTCG AAAAGTAGCC AAAGGGCCCA GCATCTGGCA TCTGCAATCC ACTTTGAACA
CP048241.1   GAAGTACCCC CGGACCGAGC TTGCTCTTCG GCCTGGCTCG AAAAGTAGCC AAAGGGCCCG GCATCTGGCA TCTGCAATCC ACTTTGAACA
CP048241.1   GAAGTACCCC CGGACCGAGC TTGCTCTTCG GCCTGGCTCG AAAAGTAGCC AAAGGGCCCG GCATCTGGCA TCTGCAATCC ACTTTGAACA
NgNTS2-F     --AGTACCCC CGGACCGAGC TT-------- ---------- ---------- ---------- ---------- ---------- ----------
NgNTS2-R     ---------- ---------- ---------- ---------- ---------- ---------- ---------- ---------- ----------

             ....|....| ....|....| ....|....| ....|....| ....|....| ....|....| ....|....| ....|....| ....|....|
                   95        105        115        125        135        145        155        165        175
LT837783.1   GCTCGAGGCC TCCCCGTCCT GCTCCAGCAG CAACACGCGA GCCTCAGGCC CAGATCCGGC CCTCGAAATC CCAGGGTCGC CCGACACTTT
```

International Journal of *Molecular Sciences*

```
CP048130.1   GCTCGAGGCC TCCCCGTCCT GCTCCAGCAG CAACACGCGA GCCTCAGGCC CAGATCCGGC CCTCGAAATC CCAGGGTCGC CCGACACTTT
CP048130.1   GCTCGAGGCC TCCCCGTCCT GCTCCAGCAG CAACACGCGA GCCTCAGGCC CAGATCCGGC CCTCGAAATC CCAGGGTCGC CCGACACTTT
CP048130.1   GCTCGAGGCC TCCCCGTCCT GCTCCAGCAG CAACACGCGA GCCTCAGGCC CAGATCCGGC CCTCGAAATC CCAGGGTCGC CCGACACTTT
CP048129.1   GCTCGAGGCC TCCCCGTCCT GCTCCAGCAG CAACACGCGA GCCTCAGGCC CAGATCCGGC CCTCGAAATC CCAGGGTCGC CCGACACTTT
CP048129.1   GCTCGAGGCC TCCCCGTCCT GCTCCAGCAG CAACACGCGA GCCTCAGGCC CAGATCCGGC CCTCGAAATC CCAGGGTCGC CCGACACTTT
CP048129.1   GCTCGAGGCC TCCCCGTCCT GCTCCAGCAG CAACACGCGA GCCTCAGGCC CAGATCCGGC CCTCGAAATC CCAGGGTCGC CCGACACTTT
CP089623.1   GCTCGAGGCC TCCCCGTCCT GCTCCAGCAG CAACACGCGA GCCTCAGGCC CAGATCCGGC CCTCGAAATC CCAGGGTCGC CCGACACTTT
CP089623.1   GCTCGAGGCC TCCCCGTCCT GCTCCAGCAG CAACACGCGA GCCTCAGGCC CAGATCCGGC CCTCGAAATC CCAGGGTCGC CCGACACTTT
LT837773.1   GCTCGAGGCC TCCCCGTCCT GCTCCAGCAG CAACACGCGA GCCTCAGGCC CAGATCCGGC CCTCGAAATC CCAGGGTCGC CCGACACTTT
LT837755.1   GCTCGAGGCC TCCCCGTCCT GCTCCAGCAG CAACACGCGA GCCTCAGGCC CAGATCCGGC CCTCGAAATC CCAGGGTCGC CCGACACTTT
CR380958.2   GCTCGAGGCC TCCCCGTCCT GCTCCAGCAG CAACACGCGA GCCTCAGGCC CAGATCCGGC CCTCGAAATC CCAGGGTCGC CCGACACTTT
LT837789.1   GCTCGAGGCC TCCCCGTCCT GCTCCAGCAG CAACACGCGA GCCTCAGGCC CAGATCCGGC CCTCGAAATC CCAGGGTCGC CCGACACTTT
LT837788.1   GCTCGAGGCC TCCCCGTCCT GCTCCAGCAG CAACACGCGA GCCTCAGGCC CAGATCCGGC CCTCGAAATC CCAGGGTCGC CCGACACTTT
LT837787.1   GCTCGAGGCC TCCCCGTCCT GCTCCAGCAG CAACACGCGA GCCTCAGGCC CAGATCCGGC CCTCGAAATC CCAGGGTCGC CCGACACTTT
LT837781.1   GCTCGAGGCC TCCCCGTCCT GCTCCAGCAG CAACACGCGA GCCTCAGGCC CAGATCCGGC CCTCGAAATC CCAGGGTCGC CCGACACTTT
LT837776.1   GCTCGAGGCC TCCCCGTCCT GTCCGAGCAG CAACACGCGA GCCTCAGGCC CAGATCCGGC CCTCGAAATC CCAGGGTCGC CCGACACTTT
LT837782.1   GCTCGAGGCC TCCCCGTCCT GCTCCAGCAG CAACACGCGA GCCCCAGGCC CAGATCCGGC CCTCGAAATC CCAGGGTCGC CCGACACTTT
LT837780.1   GCTCGAGGCC TCCCCGTCCT GCTCCAGCAG CAACACGCGA GCCTCAGGCC CAGATCCGGC CCTCGAAATC CCAGGGTCGC CCGACACTTT
LT837775.1   GCTCGAGGCC TCCCCGTCCT GTCCGAGCAG CAACACGCGA GCCTCAGGCC CAGATCCGGC CCTCGAAATC CCAGGGTCGC CCGACACTTT
LT837753.1   GCTCGAGGCC TCCCCGTCCT GCTCCAGCAG CAACACGCGA GCCTCAGGCC CAGATCCGGC CCTCGAAATC CCAGGGTCGC CCGACACTTT
FN554379.1   GCTCGAGGCC TCCCCGTCCT GCTCCAGCAG CAACACGCGA GCCTCAGGCC CAGATCCGGC CCTCGAAATC CCAGGGTCGC CCGACACTTT
LT837754.1   GCTCGAGGCC TCCCCGTCCT GCTCCAGCAG CAACACGCGA GCCTCAGGCC CAGATCCGGC CCTCGAAATC CCAGGGTCGC CCGACACTTT
CP089636.1   GCTCGAGGCC TCCCCGTCCT GCTCCAGCAG CAACACGCGA GCCTCAGGCC CAGATCCGGC CCTCGAAATC CCAGGGTCGC CCGACACTTT
CP089636.1   GCTCGAGGCC TCCCCGTCCT GCTCCAGCAG CAACACGCGA GCCTCAGGCC CAGATCCGGC CCTCGAAATC CCAGGGTCGC CCGACACTTT
CP089663.1   GCTCGAGGCC TCCCCGTCCT GCTCCAGCAG CAACACGCGA GCCTCAGGCC CAGATCCGGC CCTCGAAATC CCAGGGTCGC CCGACACTTT
LT837772.1   GCTCGAGGCC TCCCCGTCCT GCTCCAGCAG CAACACGCGA GCCTCAGGCC CAGATCCGGC CCTCGAAATC CCAGGGTCGC CCGACACTTT
LT837757.1   GCTCGAGGCC TCCCCGTCCT GCTCCAGCAG CAACACGCGA GCCTCAGGCC CAGATCCGGC CCTCGAAATC CCAGGGTCGC CCGACACTTT
LT837762.1   GCTCGAGGCC TCCCCGTCCT GCTCCAGCAG CAACACGCGA GCCTCAGGCC CAGATCCGGC CCTCGAAATC CCAGGGTCGC CCGACACTTT
LT837761.1   GCTCGAGGCC TCCCCGTCCT GCTCCAGCAG CAACACGCGA GCCTCAGGCC CAGATCCGGC CCTCGAAATC CCAGGGTCGC CCGACACTTT
CP089637.1   GCTCGAGGCC TCCCCGTCCT GCTCCAGCAG CAACACGCGA GCCTCAGGCC CAGATCCGGC CCTCGAAATC CCAGGGTCGC CCGACACTTT
CP089637.1   GCTCGAGGCC TCCCCGTCCT GCTCCAGCAG CAACACGCGA GCCTCAGGCC CAGATCCGGC CCTCGAAATC CCAGGGTCGC CCGACACTTT
LT837758.1   GCTCGAGGCC TCCCCGTCCT GCTCCAGCAG CAACACGCGA GCCTCAGGCC CAGATCCGGC CCTCGAAATC CCAGGGTCGC CCGACACTTT
CP089715.1   GCTCGAGGCC TCCCCGTCCT GCTCCAGCAG CAACACGCGA GCCTCAGGCC CAGATCCGGC CCTCGAAATC CCAGGGTCGC CCGACACTTT
CP089715.1   GCTCGAGGCC TCCCCGTCCT GCTCCAGCAG CAACACGCGA GCCTCAGGCC CAGATCCGGC CCTCGAAATC CCAGGGTCGC CCGACACTTT
CP089715.1   GCTCGAGGCC TCCCCGTCCT GCTCCAGCAG CAACACGCGA GCCTCAGGCC CAGATCCGGC CCTCGAAATC CCAGGGTCGC CCGACACTTT
LT837767.1   GCTCGAGGCC TCCCCGTCCT GCTCCAGCAG CAACACGCGA GCCTCAGGCC CAGATCCGGC CCTCGAAATC CCAGGGTCGC CCGACACTTT
LT837765.1   GCTCGAGGCC TCCCCGTCCT GCTCCAGCAG CAACACGCGA GCCTCAGGCC CAGATCCGGC CCTCGAAATC CCAGGGTCGC CCGACACTTT
CP089662.1   GCTCGAGGCC TCCCCGTCCT GCTCCAGCAG CAACACGCGA GCCTCAGGCC CAGATCCGGC CCTCGAAATC CCAGGGTCGC CCGACACTTT
CP089662.1   GCTCGAGGCC TCCCCGTCCT GCTCCAGCAG CAACACGCGA GCCTCAGGCC CAGATCCGGC CCTCGAAATC CCAGGGTCGC CCGACACTTT
LT837790.1   GCTCGAGGCC TCCCCGTCCT GCTCCAGCAG CAACACGCGA GCCTCAGGCC CAGATCCGGC CCTCGAAATC CCAGGGTCGC CCGACACTTT
LT837774.1   GCTCGAGGCC TCCCCGTCCT GTCCGAGCAG CAACACGCGA GCCTCAGGCC CAGATCCGGC CCTCGAAATC CCAGGGTCGC CCGACACTTT
LT837751.1   GCTCGAGGCC TCCCCGTCCT GCTCCAGCAG CAACACGCGA GCCTCAGGCC CAGATCCGGC CCTCGAAATC CCAGGGTCGC CCGACACTTT
```

International Journal of *Molecular Sciences*

MDPI

```
CP048242.1    GCTCGAGGCC TCCCCGTCCT GCTCCAGCAG CAACACGCGA GCCTCAGGCC CAGATCCGGC CCTCGAAATC CCAGGGTCGC CCGACACTTT
CP048242.1    GCTCGAGGCC TCCCCGTCCT GCTCCAGCAG CAACACGCGA GCCTCAGGCC CAGATCCGGC CCTCGAAATC CCAGGGTCGC CCGACACTTT
CP048241.1    GCTCGAGGCC TCCCCGTCCT GTCCGAGCAG CAACACGCGA GCCTCAGGCC CAGATCCGGC CCTCGAAATC CCAGGGTCGC CCGACACTTT
CP048241.1    GCTCGAGGCC TCCCCGTCCT GTCCGAGCAG CAACACGCGA GCCTCAGGCC CAGATCCGGC CCTCGAAATC CCAGGGTCGC CCGACACTTT
NgNTS2-F      ---------- ---------- ---------- ---------- ---------- ---------- ---------- ---------- ----------
NgNTS2-R      ---------- ---------- ---------- ---------- ---------- ---------- ---------- ---------- ----------

              ....|....| ....|....| ....|....| ....|....| ....|....| ....|....| ....|....| ....|....| ....|....|
                   185        195        205        215        225        235        245        255        265
LT837783.1    GCGAGCCTCC CACAGGTGCT GTCCCCAGAA ACGCCCGCAC AGCCACGCCC TTAACTATGG CTTTGGGCCC CGGCACTCGG CACTCGGCTG
CP048130.1    GCGAGCCTCC CACAGGTGCT GTCCCCAGAA ACGCCCGCAC AACCACGCCC TTAACTATGG CTTTGGGCCC CGGCACTCGG CACTCGGCTG
CP048130.1    GCGAGCCTCC CACAGGTGCT GTCCCCAGAA ACGCCCGCAC AACCACGCCC TTAACTATGG CTTTGGGCCC CGGCACTCGG CACTCGGCTG
CP048130.1    GCGAGCCTCC CACAGGTGCT GTCCCCAGAA ACGCCCGCAC AACCACGCCC TTAACTATGG CTTTGGGCCC CGGCACTCGG CACTCGGCTG
CP048129.1    GCGAGCCTCC CACAGGTGCT GTCCCCAGAA ACGCCCGCAC AACCACGCCC TTAACTATGG CTTTGGGCCC CGGCACTCGG CACTCGGCTG
CP048129.1    GCGAGCCTCC CACAGGTGCT GTCCCCAGAA ACGCCCGCAC AACCACGCCC TTAACTATGG CTTTGGGCCC CGGCACTCGG CACTCGGCTG
CP048129.1    GCGAGCCTCC CACAGGTGCT GTCCCCAGAA ACGCCCGCAC AACCACGCCC TTAACTATGG CTTTGGGCCC CGGCACTCGG CACTCGGCTG
CP089623.1    GCGAGCCTCC CACAGGTGCT GTCCCCAGAA ACGCCCGCAC AACCACGCCC TTAACTATGG CTTTGGGCCC CGGCACTCGG CACTCGGCTG
CP089623.1    GCGAGCCTCC CACAGGTGCT GTCCCCAGAA ACGCCCGCAC AACC--GCCC TTAACTATGG CTTTGGGCCC CGGCACTCGG CACTCGGCTG
LT837773.1    GCGAGCCTCC CACAGGTGCT GTCCCCAGAA ACGCCCGCAC AGCCACGCCC TTAACTATGG CTTTGGGCCC CGGCACTCGG CACTCGGCTG
LT837755.1    GCGAGCCTCC CACAGGTGCT GTCCCCAGAA ACGCCCGCAC AACCACGCCC TTAACTATGG CTTTGGGCCC CGGCACTCGG CACTCGGCTG
CR380958.2    GCGAGCCTCC CACAGGTGCT GTCCCCAGAA ACGCCCGCAC AACCACGCCC TTAACTATGG CTTTGGGCCC CGGCACTCGG CACTCGGCTG
LT837789.1    GCGAGCCTCC CACAGGTGCT GTCCCCAGAA ACGCCCGCAC AACCACGCCC TTAACTATGG CTTTGGGCCC CGGCACTCGG CACTCGGCTG
LT837788.1    GCGAGCCTCC CACAGGTGCT GTCCCCAGAA ACGCCCGCAC AACCACGCCC TTAACTATGG CTTTGGGCCC CGGCACTCGG CACTCGGCTG
LT837787.1    GCGAGCCTCC CACAGGTGCT GTCCCCAGAA ACGCCCGCAC AACCACGCCC TTAACTATGG CTTTGGGCCC CGGCACTCGG CACTCGGCTG
LT837781.1    GCGAGCCTCC CACAGGTGCT GTCCCCAGAA ACGCCCGCAC AACCACGCCC TTAACTATGG CTTTGGGCCC CGGCACTCGG CACTCGGCTG
LT837776.1    GCGAGCCTCC CACAGGTGCT GTCCCCAGAA ACGCCCGCAC AGCCACGCCC TTAACTATGG CTTTGGGCCC CGGCACTCGG CACTCGGCTG
LT837782.1    GCGAGCCTCC CACAGGTGCT GTCCCCAGAA ACGCCCGCAC AGCCACGCCC TTAACTATGG CTTTGGGCCC CGGCACTCGG CACTCGGCTG
LT837780.1    GCGAGCCTCC CACAGGTGCT GTCCCCAGAA ACGCCCGCAC AACCACGCCC TTAACTATGG CTTTGGGCCC CGGCACTCGG CACTCGGCTG
LT837775.1    GCGAGCCTCC CACAGGTGCT GTCCCCAGAA ACGCCCGCAC AGCCACGCCC TTAACTATGG CTTTGGGCCC CGGCACTCGG CACTCGGCTG
LT837753.1    GCGAGCCTCC CACAGGTGCT GTCCCCAGAA ACGCCCGCAC AACCACGCCC TTAACTATGG CTTTGGGCCC CGGCACTCGG CACTCGGCTG
FN554379.1    GCGAGCCTCC CACAGGTGCT GTCCCCAGAA ACGCCCGCAC AACCACGCCC TTAACTATGG CTTTGGGCCC CGGCACTCGG CACTCGGCTG
LT837754.1    GCGAGCCTCC CACAGGTGCT GTCCCCAGAA ACGCCCGCAC AACCACGCCC TTAACTATGG CTTTGGGCCC CGGCACTCGG CACTCGGCTG
CP089636.1    GCGAGCCTCC CACAGGTGCT GTCCCCAGAA ACGCCCGCAC AGCCACGCCC TTAACTATGG CTTTGGGCCC CGGCACTCGG CACTCGGCTG
CP089636.1    GCGAGCCTCC CACAGGTGCT GTCCCCAGAA ACGCCCGCAC AGCCACGCCC TTAACTATGG CTTTGGGCCC CGGCACTCGG CACTCGGCTG
CP089663.1    GCGAGCCTCC CACAGGTGCT GTCCCCAGAA ACGCCCGCAC AACCACGCCC TTAACTATGG CTTTGGGCCC CGGCACTCGG CACTCGGCTG
LT837772.1    GCGAGCCTCC CACAGGTGCT GTCCCCAGAA ACGCCCGCAC AGCCACGCCC TTAACTATGG CTTTGGGCCC CGGCACTCGG CACTCGGCTG
LT837757.1    GCGAGCCTCC CACAGGTGCT GTCCCCAGAA ACGCCCGCAC AACCACGCCC TTAACTATGG CTTTGGGCCC CGGCACTCGG CACTCGGCTG
LT837762.1    GCGAGCCTCC CACAGGTGCT GTCCCCAGAA ACGCCCGCAC AGCCACGCCC TTAACTATGG CTTTGGGCCC CGGCACTCGG CACTCGGCTG
LT837761.1    GCGAGCCTCC CACAGGTGCT GTCCCCAGAA ACGCCCGCAC AGCCACGCCC TTAACTATGG CTTTGGGCCC CGGCACTCGG CACTCGGCTG
CP089637.1    GCGAGCCTCC CACAGGTGCT GTCCCCAGAA ACGCCCGCAC AGCCACGCCC TTAACTATGG CTTTGGGCCC CGGCACTCGG CACTCGGCTG
CP089637.1    GCGAGCCTCC CACAGGTGCT GTCCCCAGAA ACGCCCGCAC AGCCACGCCC TTAACTATGG CTTTGGGCCC CGGCACTCGG CACTCGGCTG
LT837758.1    GCGAGCCTCC CACAGGTGCT GTCCCCAGAA ACGCCCGCAC AGCCACGCCC TTAACTATGG CTTTGGGCCC CGGCACTCGG CACTCGGCTG
```

International Journal of
Molecular Sciences

```
CP089715.1   GCGAGCCTCC CACAGGTGCT GTCCCCAGAA ACGCCCGCAC AGCCACGCCC TTAACTATGG CTTTGGGCCC CGGCACTCGG CACTCGGCTG
CP089715.1   GCGAGCCTCC CACAGGTGCT GTCCCCAGAA ACGCCCGCAC AGCCACGCCC TTAACTATGG CTTTGGGCCC CGGCACTCGG CACTCGGCTG
CP089715.1   GCGAGCCTCC CACAGGTGCT GTCCCCAGAA ACGCCCGCAC AACCACGCCC TTAACTATGG CTTTGGGCCC CGGCACTCGG CACTCGGCTG
LT837767.1   GCGAGCCTCC CACAGGTGCT GTCCCCAGAA ACGCCCGCAC AGCCACGCCC TTAACTATGG CTTTGGGCCC CGGCACTCGG CACTCGGCTG
LT837765.1   GCGAGCCTCC CACAGGTGCT GTCCCCAGAA ACGCCCGCAC AGCCACGCCC TTAACTATGG CTTTGGGCCC CGGCACTCGG CACTCGGCTG
CP089662.1   GCGAGCCTCC CACAGGTGCT GTCCCCAGAA ACGCCCGCAC AACCACGCCC TTAACTATGG CTTTGGGCCC CGGCACTCGG CACTCGGCTG
CP089662.1   GCGAGCCTCC CACAGGTGCT GTCCCCAGAA ACGCCCGCAC AACCACGCCC TTAACTATGG CTTTGGGCCC CGGCACTCGG CACTCGGCTG
LT837790.1   GCGAGCCTCC CACAGGTGCT GTCCCCAGAA ACGCCCGCAC AACCACGCCC TTAACTATGG CTTTGGGCCC CGGCACTCGG CACTCGGCTG
LT837774.1   GCGAGCCTCC CACAGGTGCT GTCCCCAGAA ACGCCCGCAC AGCCACGCCC TTAACTATGG CTTTGGGCCC CGGCACTCGG CACTCGGCTG
LT837751.1   GCGAGCCTCC CACAGGTGCT GTCCCCAGAA ACGCCCGCAC AACCACGCCC TTAACTATGG CTTTGGGCCC CGGCACTCGG CACTCGGCTG
CP048242.1   GCGAGCCTCC CACAGGTGCT GTCCCCAGAA ACGCCCGCAC AACCACGCCC TTAACTATGG CTTTGGGCCC CGGCACTCGG CACTCGGCTG
CP048242.1   GCGAGCCTCC CACAGGTGCT GTCCCCAGAA ACGCCCGCAC AGCCACGCCC TTAACTATGG CTTTGGGCCC CGGCACTCGG CACTCGGCTG
CP048241.1   GCGAGCCTCC CACAGGTGCT GTCCCCAGAA ACGCCCGCAC AGCCACGCCC TTAACTATGG CTTTGGGCCC CGGCACTCGG CACTCGGCTG
CP048241.1   GCGAGCCTCC CACAGGTGCT GTCCCCAGAA ACGCCCGCAC AGCCACGCCC TTAACTATGG CTTTGGGCCC CGGCACTCGG CACTCGGCTG
NgNTS2-F     ---------- ---------- ---------- ---------- ---------- ---------- ---------- ---------- ----------
NgNTS2-R     ---------- ---------- ---------- ---------- ---------- ---------- ---------- ---------- ----------

             ....|....| ....|....| ....|....| ....|....| ....|....| ....|....| ....|....| ....|....| ....|....|
                  275        285        295        305        315        325        335        345        355
LT837783.1   CGAAAAATAA AACGTGTGCC GTCGCACGTG GTCACATGGC CGCCCGCGCA CTCCGCACGT CGCCCCGCCC CCGGATTTCC GCCCATGACT
CP048130.1   CGAAAAATAA AACGTGTGCC GTCGCACGTG GTCACATGGC CGCCCGCGCA CTCCGCACGT CGCCCCGCCC CCGGATTTCC GCCCATGACT
CP048130.1   CGAAAAATAA AACGTGTGCC GTCGCACGTG GTCACATGGC CGCCCGCGCA CTCCGCACGT CGCCCCGCCC CCGGATTTCC GCCCATGACT
CP048130.1   CGAAAAATAA AACGTGTGCC GTCGCACGTG GTCACATGGC CGCCCGCGCA CTCCGCACGT CGCCCCGCCC CCGGATTTCC GCCCATGACT
CP048129.1   CGAAAAATAA AACGTGTGCC GTCGCACGTG GTCACATGGC CGCCCGCGCA CTCCGCACGT CGCCCCGCCC CCGGATTTCC GCCCATGACT
CP048129.1   CGAAAAATAA AACGTGTGCC GTCGCACGTG GTCACATGGC CGCCCGCGCA CTCCGCACGT CGCCCCGCCC CCGGATTTCC GCCCATGACT
CP048129.1   CGAAAAATAA AACGTGTGCC GTCGCACGTG GTCACATGGC CGCCCGCGCA CTCCGCACGT CGCCCCGCCC CCGGATTTCC GCCCATGACT
CP089623.1   CGAAAAATAA AACGTGTGCC GTCGCACGTG GTCACATGGC CGCCCGCGCA CTCCGCACGT CGCCCCGCCC CCGGATTTCC GCCCATGACT
CP089623.1   CGAAAAATAA AACGTGTGCC GTCGCACGTG GTCACATGGC CGCCCGCGCA CTCCGCACGT CGCCCCCGCC CCCGATTTCC GCC-ATGACT
LT837773.1   CGAAAAATAA AACGTGTGCC GTCGCACGTG GTCACATGGC CGCCCGCGCA CTCCGCACGT CGCCCCGCCC CCGGATTTCC GCCCATGACT
LT837755.1   CGAAAAATAA AACGTGTGCC GTCGCACGTG GTCACATGGC CGCCCGCGCA CTCCGCACGT CGCCCCGCCC CCGGATTTCC GCCCATGACT
CR380958.2   CGAAAAATAA AACGTGTGCC GTCGCACGTG GTCACATGGC CGCCCGCGCA CTCCGCACGT CGCCCCGCCC CCGGATTTCC GCCCATGACT
LT837789.1   CGAAAAATAA AACGTGTGCC GTCGCACGTG GTCACATGGC CGCCCGCGCA CTCCGCACGT CGCCCCGCCC CCGGATTTCC GCCCATGACT
LT837788.1   CGAAAAATAA AACGTGTGCC GTCGCACGTG GTCACATGGC CGCCCGCGCA CTCCGCACGT CGCCCCGCCC CCGGATTTCC GCCCATGACT
LT837787.1   CGAAAAATAA AACGTGTGCC GTCGCACGTG GTCACATGGC CGCCCGCGCA CTCCGCACGT CGCCCCGCCC CCGGATTTCC GCCCATGACT
LT837781.1   CGAAAAATAA AACGTGTGCC GTCGCACGTG GTCACATGGC CGCCCGCGCA CTCCGCACGT CGCCCCGCCC CCGGATTTCC GCCCATGACT
LT837776.1   CGAAAAATAA AACGTGTGCC GTCGCACGTG GTCACATGGC CGCCCGCGCA CTCCGCACGT CGCCCCGCCC CCGGATTTCC GCCCATGACT
LT837782.1   CGAAAAATAA AACGTGTGCC GTCGCACGTG GTCACATGGC CGCCCGCGCA CTCCGCACGT CGCCCCGCCC CCGGATTTCC GCCCATGACT
LT837780.1   CGAAAAATAA AACGTGTGCC GTCGCACGTG GTCACATGGC CGCCCGCGCA CTCCGCACGT CGCCCCGCCC CCGGATTTCC GCCCATGACT
LT837775.1   CGAAAAATAA AACGTGTGCC GTCGCACGTG GTCACATGGC CGCCCGCGCA CTCCGCACGT CGCCCCGCCC CCGGATTTCC GCCCATGACT
LT837753.1   CGAAAAATAA AACGTGTGCC GTCGCACGTA GTCACATGGC CGCCCGCGCA CTCCGCACGT CGCCCCGCCC CCGGATTTCC GCCCATGACT
FN554379.1   CGAAAAATAA AACGTGTGCC GTCGCACGTG GTCACATGGC CGCCCGCGCA CTCCGCACGT CGCCCCGCCC CCGGATTTCC GCCCATGACT
LT837754.1   CGAAAAATAA AACGTGTGCC GTCGCACGTG GTCACATGGC CGCCCGCGCA CTCCGCACGT CGCCCCGCCC CCGGATTTCC GCCCATGACT
```

```
CP089636.1    CGAAAAATAA AACGTGTGCC GTCGCACGTG GTCACATGGC CGCCCGCGCA CTCCGCACGT CGCCCCGCCC CCGGATTTCC GCCCATGACT
CP089636.1    CGAAAAATAA AACGTGTGCC GTCGCACGTG GTCACATGGC CGCCCGCGCA CTCCGCACGT CGCCCCGCCC CCGGATTTCC GCCCATGACT
CP089663.1    CGAAAAATAA AACGTGTGCC GTCGCACGTG GTCACATGGC CGCCCGCGCA CTCCGCACGT CGCCCCGCCC CCGGATTTCC GCCCATGACT
LT837772.1    CGAAAAATAA AACGTGTGCC GTCGCACGTG GTCACATGGC CGCCCGCGCA CTCCGCACGT CGCCCCGCCC CCGGATTTCC GCCCATGACT
LT837757.1    CGAAAAATAA AACGTGTGCC GTCGCACGTG GTCACATGGC CGCCCGCGCA CTCCGCACGT CGCCCCGCCC CCGGATTTCC GCCCATGACT
LT837762.1    CGAAAAATAA AACGTGTGCC GTCGCACGTG GTCACATGGC CGCCCGCGCA CTCCGCACGT CGCCCCGCCC CCGGATTTCC GCCCATGACT
LT837761.1    CGAAAAATAA AACGTGTGCC GTCGCACGTG GTCACATGGC CGCCCGCGCA CTCCGCACGT CGCCCCGCCC CCGGATTTCC GCCCATGACT
CP089637.1    CGAAAA-TAA AACGTGTGCC GTCGCACGTG GTCACATGGC CGCCCGCGCA CTCCGCACGT CGCCCCGCCC CCGGATTTCC GCCCATGACT
CP089637.1    CGAAAA-TAA AACGTGTGCC GTCGCACGTG GTCACATGGC CGCCCGCGCA CTCCGCACGT CGCCCCGCCC CCGGATTTCC GCCCATGACT
LT837758.1    CGAAAAATAA AACGTGTGCC GTCGCACGTG GTCACATGGC CGCCCGCGCA CTCCGCACGT CGCCCCGCCC CCGGATTTCC GCCCATGACT
CP089715.1    CGAAAAATAA AACGTGTGCC GTCGCACGTG GTCACATGGC CGCCCGCGCA CTCCGCACGT CGCCCCGCCC CCGGATTTCC GCCCATGACT
CP089715.1    CGAAAAATAA AACGTGTGCC GTCGCACGTG GTCACATGGC CGCCCGCGCA CTCCGCACGT CGCCCCGCCC CCGGATTTCC GCCCATGACT
CP089715.1    CGAAAAATAA AACGTGTGCC GTCGCACGTA GTCACATGGC CGCCCGCGCA CTCCGCACGT CGCCCCGCCC CCGGATTTCC GCCCATGACT
LT837767.1    CGAAAAATAA AACGTGTGCC GTCGCACGTG GTCACATGGC CGCCCGCGCA CTCCGCACGT CGCCCCGCCC CCGGATTTCC GCCCATGACT
LT837765.1    CGAAAAATAA AACGTGTGCC GTCGCACGTG GTCACATGGC CGCCCGCGCA CTCCGCACGT CGCCCCGCCC CCGGATTTCC GCCCATGACT
CP089662.1    CGAAAAATAA AACGTGTGCC GTCGCACGTG GTCACATGGC CGCCCGCGCA CTCCGCACGT CGCCCCGCCC CCGGATTTCC GCCCATGACT
CP089662.1    CGAAAAATAA AACGTGTGCC GTCGCACGTG GTCACATGGC CGCCCGCGCA CTCCGCACGT CGCCCCGCCC CCGGATTTCC GCCCATGACT
LT837790.1    CGAAAAATAA AACGTGTGCC GTCGCACGTG GTCACATGGC CGCCCGCGCA CTCCGCACGT CGCCCCGCCC CCGGATTTCC GCCCATGACT
LT837774.1    CGAAAAATAA AACGTGTGCC GTCGCACGTG GTCACATGGC CGCCCGCGCA CTCCGCACGT CGCCCCGCCC CCGGATTTCC GCCCATGACT
LT837751.1    CGAAAAATAA AACGTGTGCC GTCGCACGTA GTCACATGGC CGCCCGCGCA CTCCGCACGT CGCCCCGCCC CCGGATTTCC GCCCATGACT
CP048242.1    CGAAAAATAA AACGTGTGCC GTCGCACGTG GTCACATGGC CGCCCGCGCA CTCCGCACGT CGCCCCGCCC CCGGATTTCC GCCCATGACT
CP048242.1    CGAAAAATAA AACGTGTGCC GTCGCACGTG GTCACATGGC CGCCCGCGCA CTCCGCACGT CGCCC-GCCC CCGGATTTCC GCCCATGACT
CP048241.1    CGAAAAATAA AACGTGTGCC GTCGCACGTG GTCACATGGC CGCCCGCGCA CTCCGCACGT CGCCCCGCCC CCGGATTTCC GCCCATGACT
CP048241.1    CGAAAAATAA AACGTGTGCC GTCGCACGTG GTCACATGGC CGCCCGCGCA CTCCGCACGT CGCCCCGCCC CCGGATTTCC GCCCATGACT
NgNTS2-F      ---------- ---------- ---------- ---------- ---------- ---------- ---------- ---------- ----------
NgNTS2-R      --------AA AACGTGTGCC GTCGCACG-- ---------- ---------- ---------- ---------- ---------- ----------
```

(e) Diagrammatic alignment representation of CauITS1 forward and reverse primers and *Candida auris* ITS1 region (partial) of rDNA locus. Computed by Multiple Sequence Alignment ClustalW algorithim.

```
              ....|....| ....|....| ....|....| ....|....| ....|....| ....|....| ....|....| ....|....| ....|....|
                   5          15         25         35         45         55         65         75         85
MK981227.1    TCCGTAGGTG AACCTGCGGA AGGATCATTA TTGATATTTT GCATACACAC TGATTTGGAT TTTAAAACTA ACCCAACGTT AAGTTCAACT
MK367811.1    TCCGTAGGTG AACCTGCGGA AGGATCATTA TTGATATTTT GCATACACAC TGATTTGGAT TTTAAAACTA ACCCAACGTT AAGTTCAACT
MH071441.1    TCCGTAGGTG AACCTGCGGA AGGATCATTA TTGATATTTT GCATACACAC TGATTTGGAT TTTAAAACTA ACCCAACGTT AAGTTCAACT
MH118269.2    TCCGTAGGTG AACCTGCGGA AGGATCATTA TTGATATTTT GCATACACAC TGATTTGGAT TTTAAAACTA ACCCAACGTT AAGTTCAACT
MH174460.1    TCCGTAGGTG AACCTGCGGA AGGATCATTA TTGATATTTT GCATACACAC TGATTTGGAT TTTAAAACTA ACCCAACGTT AAGTTCAACT
CP163329.1    TCCGTAGGTG AACCTGCGGA AGGATCATTA TTGATATTTT GCATACACAC TGATTTGGAT TTTAAAACTA ACCCAACGTT AAGTTCAACT
CP163322.1    TCCGTAGGTG AACCTGCGGA AGGATCATTA TTGATATTTT GCATACACAC TGATTTGGAT TTTAAAACTA ACCCAACGTT AAGTTCAACT
CP163350.1    TCCGTAGGTG AACCTGCGGA AGGATCATTA TTGATATTTT GCATACACAC TGATTTGGAT TTTAAAACTA ACCCAACGTT AAGTTCAACT
CP163357.1    TCCGTAGGTG AACCTGCGGA AGGATCATTA TTGATATTTT GCATACACAC TGATTTGGAT TTTAAAACTA ACCCAACGTT AAGTTCAACT
```

International Journal of Molecular Sciences

MDPI

CP163336.1	TCCGTAGGTG	AACCTGCGGA	AGGATCATTA	TTGATATTTT	GCATACACAC	TGATTTGGAT	TTTAAAACTA	ACCCAACGTT	AAGTTCAACT
CP163315.1	TCCGTAGGTG	AACCTGCGGA	AGGATCATTA	TTGATATTTT	GCATACACAC	TGATTTGGAT	TTTAAAACTA	ACCCAACGTT	AAGTTCAACT
CP163364.1	TCCGTAGGTG	AACCTGCGGA	AGGATCATTA	TTGATATTTT	GCATACACAC	TGATTTGGAT	TTTAAAACTA	ACCCAACGTT	AAGTTCAACT
CP163343.1	TCCGTAGGTG	AACCTGCGGA	AGGATCATTA	TTGATATTTT	GCATACACAC	TGATTTGGAT	TTTAAAACTA	ACCCAACGTT	AAGTTCAACT
PP994477.1	TCCGTAGGTG	AACCTGCGGA	AGGATCATTA	TTGATATTTT	GCATACACAC	TGATTTGGAT	TTTAAAACTA	ACCCAACGTT	AAGTTCAACT
CP157519.1	TCCGTAGGTG	AACCTGCGGA	AGGATCATTA	TTGATATTTT	GCATACACAC	TGATTTGGAT	TTTAAAACTA	ACCCAACGTT	AAGTTCAACT
CP157517.1	TCCGTAGGTG	AACCTGCGGA	AGGATCATTA	TTGATATTTT	GCATACACAC	TGATTTGGAT	TTTAAAACTA	ACCCAACGTT	AAGTTCAACT
CP157516.1	TCCGTAGGTG	AACCTGCGGA	AGGATCATTA	TTGATATTTT	GCATACACAC	TGATTTGGAT	TTTAAAACTA	ACCCAACGTT	AAGTTCAACT
CP157512.1	TCCGTAGGTG	AACCTGCGGA	AGGATCATTA	TTGATATTTT	GCATACACAC	TGATTTGGAT	TTTAAAACTA	ACCCAACGTT	AAGTTCAACT
CP157510.1	TCCGTAGGTG	AACCTGCGGA	AGGATCATTA	TTGATATTTT	GCATACACAC	TGATTTGGAT	TTTAAAACTA	ACCCAACGTT	AAGTTCAACT
CP157509.1	TCCGTAGGTG	AACCTGCGGA	AGGATCATTA	TTGATATTTT	GCATACACAC	TGATTTGGAT	TTTAAAACTA	ACCCAACGTT	AAGTTCAACT
CP156633.1	TCCGTAGGTG	AACCTGCGGA	AGGATCATTA	TTGATATTTT	GCATACACAC	TGATTTGGAT	TTTAAAACTA	ACCCAACGTT	AAGTTCAACT
CP156631.1	TCCGTAGGTG	AACCTGCGGA	AGGATCATTA	TTGATATTTT	GCATACACAC	TGATTTGGAT	TTTAAAACTA	ACCCAACGTT	AAGTTCAACT
CP156630.1	TCCGTAGGTG	AACCTGCGGA	AGGATCATTA	TTGATATTTT	GCATACACAC	TGATTTGGAT	TTTAAAACTA	ACCCAACGTT	AAGTTCAACT
CP147453.1	TCCGTAGGTG	AACCTGCGGA	AGGATCATTA	TTGATATTTT	GCATACACAC	TGATTTGGAT	TTTAAAACTA	ACCCAACGTT	AAGTTCAACT
CP147467.1	TCCGTAGGTG	AACCTGCGGA	AGGATCATTA	TTGATATTTT	GCATACACAC	TGATTTGGAT	TTTAAAACTA	ACCCAACGTT	AAGTTCAACT
CP147446.1	TCCGTAGGTG	AACCTGCGGA	AGGATCATTA	TTGATATTTT	GCATACACAC	TGATTTGGAT	TTTAAAACTA	ACCCAACGTT	AAGTTCAACT
CP147481.1	TCCGTAGGTG	AACCTGCGGA	AGGATCATTA	TTGATATTTT	GCATACACAC	TGATTTGGAT	TTTAAAACTA	ACCCAACGTT	AAGTTCAACT
CP147439.1	TCCGTAGGTG	AACCTGCGGA	AGGATCATTA	TTGATATTTT	GCATACACAC	TGATTTGGAT	TTTAAAACTA	ACCCAACGTT	AAGTTCAACT
CP147460.1	TCCGTAGGTG	AACCTGCGGA	AGGATCATTA	TTGATATTTT	GCATACACAC	TGATTTGGAT	TTTAAAACTA	ACCCAACGTT	AAGTTCAACT
CP147474.1	TCCGTAGGTG	AACCTGCGGA	AGGATCATTA	TTGATATTTT	GCATACACAC	TGATTTGGAT	TTTAAAACTA	ACCCAACGTT	AAGTTCAACT
CP147432.1	TCCGTAGGTG	AACCTGCGGA	AGGATCATTA	TTGATATTTT	GCATACACAC	TGATTTGGAT	TTTAAAACTA	ACCCAACGTT	AAGTTCAACT
CP126633.2	TCCGTAGGTG	AACCTGCGGA	AGGATCATTA	TTGATATTTT	GCATACACAC	TGATTTGGAT	TTTAAAACTA	ACCCAACGTT	AAGTTCAACT
CP126454.2	TCCGTAGGTG	AACCTGCGGA	AGGATCATTA	TTGATATTTT	GCATACACAC	TGATTTGGAT	TTTAAAACTA	ACCCAACGTT	AAGTTCAACT
CP126453.2	TCCGTAGGTG	AACCTGCGGA	AGGATCATTA	TTGATATTTT	GCATACACAC	TGATTTGGAT	TTTAAAACTA	ACCCAACGTT	AAGTTCAACT
CP126450.2	TCCGTAGGTG	AACCTGCGGA	AGGATCATTA	TTGATATTTT	GCATACACAC	TGATTTGGAT	TTTAAAACTA	ACCCAACGTT	AAGTTCAACT
OP626776.1	TCCGTAGGTG	AACCTGCGGA	AGGATCATTA	TTGATATTTT	GCATACACAC	TGATTTGGAT	TTTAAAACTA	ACCCAACGTT	AAGTTCAACT
CP133934.1	TCCGTAGGTG	AACCTGCGGA	AGGATCATTA	TTGATATTTT	GCATACACAC	TGATTTGGAT	TTTAAAACTA	ACCCAACGTT	AAGTTCAACT
CP133933.1	TCCGTAGGTG	AACCTGCGGA	AGGATCATTA	TTGATATTTT	GCATACACAC	TGATTTGGAT	TTTAAAACTA	ACCCAACGTT	AAGTTCAACT
CP133932.1	TCCGTAGGTG	AACCTGCGGA	AGGATCATTA	TTGATATTTT	GCATACACAC	TGATTTGGAT	TTTAAAACTA	ACCCAACGTT	AAGTTCAACT
OQ581776.1	TCCGTAGGTG	AACCTGCGGA	AGGATCATTA	TTGATATTTT	GCATACACAC	TGATTTGGAT	TTTAAAACTA	ACCCAACGTT	AAGTTCAACT
OQ513465.1	TCCGTAGGTG	AACCTGCGGA	AGGATCATTA	TTGATATTTT	GCATACACAC	TGATTTGGAT	TTTAAAACTA	ACCCAACGTT	AAGTTCAACT
OQ513462.1	TCCGTAGGTG	AACCTGCGGA	AGGATCATTA	TTGATATTTT	GCATACACAC	TGATTTGGAT	TTTAAAACTA	ACCCAACGTT	AAGTTCAACT
KP326583.1	TCCGTAGGTG	AACCTGCGGA	AGGATCATTA	TTGATATTTT	GCATACACAC	TGATTTGGAT	TTTAAAACTA	ACCCAACGTT	AAGTTCAACT
KP131674.1	TCCGTAGGTG	AACCTGCGGA	AGGATCATTA	TTGATATTTT	GCATACACAC	TGATTTGGAT	TTTAAAACTA	ACCCAACGTT	AAGTTCAACT
OW988727.1	TCCGTAGGTG	AACCTGCGGA	AGGATCATTA	TTGATATTTT	GCATACACAC	TGATTTGGAT	TTTAAAACTA	ACCCAACGTT	AAGTTCAACT
OW988598.1	TCCGTAGGTG	AACCTGCGGA	AGGATCATTA	TTGATATTTT	GCATACACAC	TGATTTGGAT	TTTAAAACTA	ACCCAACGTT	AAGTTCAACT
OM530139.1	TCCGTAGGTG	AACCTGCGGA	AGGATCATTA	TTGATATTTT	GCATACACAC	TGATTTGGAT	TTTAAAACTA	ACCCAACGTT	AAGTTCAACT
CP076751.1	TCCGTAGGTG	AACCTGCGGA	AGGATCATTA	TTGATATTTT	GCATACACAC	TGATTTGGAT	TTTAAAACTA	ACCCAACGTT	AAGTTCAACT
CP077033.1	TCCGTAGGTG	AACCTGCGGA	AGGATCATTA	TTGATATTTT	GCATACACAC	TGATTTGGAT	TTTAAAACTA	ACCCAACGTT	AAGTTCAACT
CP076991.1	TCCGTAGGTG	AACCTGCGGA	AGGATCATTA	TTGATATTTT	GCATACACAC	TGATTTGGAT	TTTAAAACTA	ACCCAACGTT	AAGTTCAACT
CauITS1-F	----------	----------	----------	----------	----------	------GGAT	TTTAAAACTA	ACCCAACG--	----------

International Journal of *Molecular Sciences*

MDPI

```
CauITS1-R     ---------- ---------- ---------- ---------- ---------- ---------- ---------- ---------- ----------

              ....|....| ....|....| ....|....| ....|....| ....|....| ....|....| ....|....| ....|....| ....|....|
                      95        105        115        125        135        145        155        165        175
MK981227.1    AAAACAAAAA CATAAAACTT TCAACAACGG ATCTCTTGGT TCTCGCATCG ATGAAGAACG CAGCGAAATG CGATACGTAG TATGACTTGC
MK367811.1    AAAACAAAAA CATAAAACTT TCAACAACGG ATCTCTTGGT TCTCGCATCG ATGAAGAACG CAGCGAAATG CGATACGTAG TATGACTTGC
MH071441.1    AAAACAAAAA CATAAAACTT TCAACAACGG ATCTCTTGGT TCTCGCATCG ATGAAGAACG CAGCGAAATG CGATACGTAG TATGACTTGC
MH118269.2    AAAACAAAAA CATAAAACTT TCAACAACGG ATCTCTTGGT TCTCGCATCG ATGAAGAACG CAGCGAAATG CGATACGTAG TATGACTTGC
MH174460.1    AAAACAAAAA CATAAAACTT TCAACAACGG ATCTCTTGGT TCTCGCATCG ATGAAGAACG CAGCGAAATG CGATACGTAG TATGACTTGC
CP163329.1    AAAACAAAAA CATAAAACTT TCAACAACGG ATCTCTTGGT TCTCGCATCG ATGAAGAACG CAGCGAAATG CGATACGTAG TATGACTTGC
CP163322.1    AAAACAAAAA CATAAAACTT TCAACAACGG ATCTCTTGGT TCTCGCATCG ATGAAGAACG CAGCGAAATG CGATACGTAG TATGACTTGC
CP163350.1    AAAACAAAAA CATAAAACTT TCAACAACGG ATCTCTTGGT TCTCGCATCG ATGAAGAACG CAGCGAAATG CGATACGTAG TATGACTTGC
CP163357.1    AAAACAAAAA CATAAAACTT TCAACAACGG ATCTCTTGGT TCTCGCATCG ATGAAGAACG CAGCGAAATG CGATACGTAG TATGACTTGC
CP163336.1    AAAACAAAAA CATAAAACTT TCAACAACGG ATCTCTTGGT TCTCGCATCG ATGAAGAACG CAGCGAAATG CGATACGTAG TATGACTTGC
CP163315.1    AAAACAAAAA CATAAAACTT TCAACAACGG ATCTCTTGGT TCTCGCATCG ATGAAGAACG CAGCGAAATG CGATACGTAG TATGACTTGC
CP163364.1    AAAACAAAAA CATAAAACTT TCAACAACGG ATCTCTTGGT TCTCGCATCG ATGAAGAACG CAGCGAAATG CGATACGTAG TATGACTTGC
CP163343.1    AAAACAAAAA CATAAAACTT TCAACAACGG ATCTCTTGGT TCTCGCATCG ATGAAGAACG CAGCGAAATG CGATACGTAG TATGACTTGC
PP994477.1    AAAACAAAAA CATAAAACTT TCAACAACGG ATCTCTTGGT TCTCGCATCG ATGAAGAACG CAGCGAAATG CGATACGTAG TATGACTTGC
CP157519.1    AAAACAAAAA CATAAAACTT TCAACAACGG ATCTCTTGGT TCTCGCATCG ATGAAGAACG CAGCGAAATG CGATACGTAG TATGACTTGC
CP157517.1    AAAACAAAAA CATAAAACTT TCAACAACGG ATCTCTTGGT TCTCGCATCG ATGAAGAACG CAGCGAAATG CGATACGTAG TATGACTTGC
CP157516.1    AAAACAAAAA CATAAAACTT TCAACAACGG ATCTCTTGGT TCTCGCATCG ATGAAGAACG CAGCGAAATG CGATACGTAG TATGACTTGC
CP157512.1    AAAACAAAAA CATAAAACTT TCAACAACGG ATCTCTTGGT TCTCGCATCG ATGAAGAACG CAGCGAAATG CGATACGTAG TATGACTTGC
CP157510.1    AAAACAAAAA CATAAAACTT TCAACAACGG ATCTCTTGGT TCTCGCATCG ATGAAGAACG CAGCGAAATG CGATACGTAG TATGACTTGC
CP157509.1    AAAACAAAAA CATAAAACTT TCAACAACGG ATCTCTTGGT TCTCGCATCG ATGAAGAACG CAGCGAAATG CGATACGTAG TATGACTTGC
CP156633.1    AAAACAAAAA CATAAAACTT TCAACAACGG ATCTCTTGGT TCTCGCATCG ATGAAGAACG CAGCGAAATG CGATACGTAG TATGACTTGC
CP156631.1    AAAACAAAAA CATAAAACTT TCAACAACGG ATCTCTTGGT TCTCGCATCG ATGAAGAACG CAGCGAAATG CGATACGTAG TATGACTTGC
CP156630.1    AAAACAAAAA CATAAAACTT TCAACAACGG ATCTCTTGGT TCTCGCATCG ATGAAGAACG CAGCGAAATG CGATACGTAG TATGACTTGC
CP147453.1    AAAACAAAAA CATAAAACTT TCAACAACGG ATCTCTTGGT TCTCGCATCG ATGAAGAACG CAGCGAAATG CGATACGTAG TATGACTTGC
CP147467.1    AAAACAAAAA CATAAAACTT TCAACAACGG ATCTCTTGGT TCTCGCATCG ATGAAGAACG CAGCGAAATG CGATACGTAG TATGACTTGC
CP147446.1    AAAACAAAAA CATAAAACTT TCAACAACGG ATCTCTTGGT TCTCGCATCG ATGAAGAACG CAGCGAAATG CGATACGTAG TATGACTTGC
CP147481.1    AAAACAAAAA CATAAAACTT TCAACAACGG ATCTCTTGGT TCTCGCATCG ATGAAGAACG CAGCGAAATG CGATACGTAG TATGACTTGC
CP147439.1    AAAACAAAAA CATAAAACTT TCAACAACGG ATCTCTTGGT TCTCGCATCG ATGAAGAACG CAGCGAAATG CGATACGTAG TATGACTTGC
CP147460.1    AAAACAAAAA CATAAAACTT TCAACAACGG ATCTCTTGGT TCTCGCATCG ATGAAGAACG CAGCGAAATG CGATACGTAG TATGACTTGC
CP147474.1    AAAACAAAAA CATAAAACTT TCAACAACGG ATCTCTTGGT TCTCGCATCG ATGAAGAACG CAGCGAAATG CGATACGTAG TATGACTTGC
CP147432.1    AAAACAAAAA CATAAAACTT TCAACAACGG ATCTCTTGGT TCTCGCATCG ATGAAGAACG CAGCGAAATG CGATACGTAG TATGACTTGC
CP126633.2    AAAACAAAAA CATAAAACTT TCAACAACGG ATCTCTTGGT TCTCGCATCG ATGAAGAACG CAGCGAAATG CGATACGTAG TATGACTTGC
CP126454.2    AAAACAAAAA CATAAAACTT TCAACAACGG ATCTCTTGGT TCTCGCATCG ATGAAGAACG CAGCGAAATG CGATACGTAG TATGACTTGC
CP126453.2    AAAACAAAAA CATAAAACTT TCAACAACGG ATCTCTTGGT TCTCGCATCG ATGAAGAACG CAGCGAAATG CGATACGTAG TATGACTTGC
CP126450.2    AAAACAAAAA CATAAAACTT TCAACAACGG ATCTCTTGGT TCTCGCATCG ATGAAGAACG CAGCGAAATG CGATACGTAG TATGACTTGC
OP626776.1    AAAACAAAAA CATAAAACTT TCAACAACGG ATCTCTTGGT TCTCGCATCG ATGAAGAACG CAGCGAAATG CGATACGTAG TATGACTTGC
CP133934.1    AAAACAAAAA CATAAAACTT TCAACAACGG ATCTCTTGGT TCTCGCATCG ATGAAGAACG CAGCGAAATG CGATACGTAG TATGACTTGC
CP133933.1    AAAACAAAAA CATAAAACTT TCAACAACGG ATCTCTTGGT TCTCGCATCG ATGAAGAACG CAGCGAAATG CGATACGTAG TATGACTTGC
```

International Journal of *Molecular Sciences*

```
CP133932.1    AAAACAAAAA CATAAAACTT TCAACAACGG ATCTCTTGGT TCTCGCATCG ATGAAGAACG CAGCGAAATG CGATACGTAG TATGACTTGC
OQ581776.1    AAAACAAAAA CATAAAACTT TCAACAACGG ATCTCTTGGT TCTCGCATCG ATGAAGAACG CAGCGAAATG CGATACGTAG TATGACTTGC
OQ513465.1    AAAACAAAAA CATAAAACTT TCAACAACGG ATCTCTTGGT TCTCGCATCG ATGAAGAACG CAGCGAAATG CGATACGTAG TATGACTTGC
OQ513462.1    AAAACAAAAA CATAAAACTT TCAACAACGG ATCTCTTGGT TCTCGCATCG ATGAAGAACG CAGCGAAATG CGATACGTAG TATGACTTGC
KP326583.1    AAAACAAAAA CATAAAACTT TCAACAACGG ATCTCTTGGT TCTCGCATCG ATGAAGAACG CAGCGAAATG CGATACGTAG TATGACTTGC
KP131674.1    AAAACAAAAA CATAAAACTT TCAACAACGG ATCTCTTGGT TCTCGCATCG ATGAAGAACG CAGCGAAATG CGATACGTAG TATGACTTGC
OW988727.1    AAAACAAAAA CATAAAACTT TCAACAACGG ATCTCTTGGT TCTCGCATCG ATGAAGAACG CAGCGAAATG CGATACGTAG TATGACTTGC
OW988598.1    AAAACAAAAA CATAAAACTT TCAACAACGG ATCTCTTGGT TCTCGCATCG ATGAAGAACG CAGCGAAATG CGATACGTAG TATGACTTGC
OM530139.1    AAAACAAAAA CATAAAACTT TCAACAACGG ATCTCTTGGT TCTCGCATCG ATGAAGAACG CAGCGAAATG CGATACGTAG TATGACTTGC
CP076751.1    AAAACAAAAA CATAAAACTT TCAACAACGG ATCTCTTGGT TCTCGCATCG ATGAAGAACG CAGCGAAATG CGATACGTAG TATGACTTGC
CP077033.1    AAAACAAAAA CATAAAACTT TCAACAACGG ATCTCTTGGT TCTCGCATCG ATGAAGAACG CAGCGAAATG CGATACGTAG TATGACTTGC
CP076991.1    AAAACAAAAA CATAAAACTT TCAACAACGG ATCTCTTGGT TCTCGCATCG ATGAAGAACG CAGCGAAATG CGATACGTAG TATGACTTGC
CauITS1-F     ---------- ---------- ---------- ---------- ---------- ---------- ---------- ---------- ----------
CauITS1-R     ---------- ---------- ---------- ---------- ---------- ---------- ---------- ---------- ----------

              ....|....| ....|....| ....|....| ....|....| ....|....| ....|....| ....|....| ....|....| ....|....|
                    185        195        205        215        225        235        245        255        265
MK981227.1    AGACGTGAAT CATCGAATCT TTGAACGCAC ATTGCGCCTT GGGGTATTCC CCAAGGCATG CCTGTTTGAG CGTGATGTCT TCTCACCAAT
MK367811.1    AGACGTGAAT CATCGAATCT TTGAACGCAC ATTGCGCCTT GGGGTATTCC CCAAGGCATG CCTGTTTGAG CGTGATGTCT TCTCACCAAT
MH071441.1    AGACGTGAAT CATCGAATCT TTGAACGCAC ATTGCGCCTT GGGGTATTCC CCAAGGCATG CCTGTTTGAG CGTGATGTCT TCTCACCAAT
MH118269.2    AGACGTGAAT CATCGAATCT TTGAACGCAC ATTGCGCCTT GGGGTATTCC CCAAGGCATG CCTGTTTGAG CGTGATGTCT TCTCACCAAT
MH174460.1    AGACGTGAAT CATCGAATCT TTGAACGCAC ATTGCGCCTT GGGGTATTCC CCAAGGCATG CCTGTTTGAG CGTGATGTCT TCTCACCAAT
CP163329.1    AGACGTGAAT CATCGAATCT TTGAACGCAC ATTGCGCCTT GGGGTATTCC CCAAGGCATG CCTGTTTGAG CGTGATGTCT TCTCACCAAT
CP163322.1    AGACGTGAAT CATCGAATCT TTGAACGCAC ATTGCGCCTT GGGGTATTCC CCAAGGCATG CCTGTTTGAG CGTGATGTCT TCTCACCAAT
CP163350.1    AGACGTGAAT CATCGAATCT TTGAACGCAC ATTGCGCCTT GGGGTATTCC CCAAGGCATG CCTGTTTGAG CGTGATGTCT TCTCACCAAT
CP163357.1    AGACGTGAAT CATCGAATCT TTGAACGCAC ATTGCGCCTT GGGGTATTCC CCAAGGCATG CCTGTTTGAG CGTGATGTCT TCTCACCAAT
CP163336.1    AGACGTGAAT CATCGAATCT TTGAACGCAC ATTGCGCCTT GGGGTATTCC CCAAGGCATG CCTGTTTGAG CGTGATGTCT TCTCACCAAT
CP163315.1    AGACGTGAAT CATCGAATCT TTGAACGCAC ATTGCGCCTT GGGGTATTCC CCAAGGCATG CCTGTTTGAG CGTGATGTCT TCTCACCAAT
CP163364.1    AGACGTGAAT CATCGAATCT TTGAACGCAC ATTGCGCCTT GGGGTATTCC CCAAGGCATG CCTGTTTGAG CGTGATGTCT TCTCACCAAT
CP163343.1    AGACGTGAAT CATCGAATCT TTGAACGCAC ATTGCGCCTT GGGGTATTCC CCAAGGCATG CCTGTTTGAG CGTGATGTCT TCTCACCAAT
PP994477.1    AGACGTGAAT CATCGAATCT TTGAACGCAC ATTGCGCCTT GGGGTATTCC CCAAGGCATG CCTGTTTGAG CGTGATGTCT TCTCACCAAT
CP157519.1    AGACGTGAAT CATCGAATCT TTGAACGCAC ATTGCGCCTT GGGGTATTCC CCAAGGCATG CCTGTTTGAG CGTGATGTCT TCTCACCAAT
CP157517.1    AGACGTGAAT CATCGAATCT TTGAACGCAC ATTGCGCCTT GGGGTATTCC CCAAGGCATG CCTGTTTGAG CGTGATGTCT TCTCACCAAT
CP157516.1    AGACGTGAAT CATCGAATCT TTGAACGCAC ATTGCGCCTT GGGGTATTCC CCAAGGCATG CCTGTTTGAG CGTGATGTCT TCTCACCAAT
CP157512.1    AGACGTGAAT CATCGAATCT TTGAACGCAC ATTGCGCCTT GGGGTATTCC CCAAGGCATG CCTGTTTGAG CGTGATGTCT TCTCACCAAT
CP157510.1    AGACGTGAAT CATCGAATCT TTGAACGCAC ATTGCGCCTT GGGGTATTCC CCAAGGCATG CCTGTTTGAG CGTGATGTCT TCTCACCAAT
CP157509.1    AGACGTGAAT CATCGAATCT TTGAACGCAC ATTGCGCCTT GGGGTATTCC CCAAGGCATG CCTGTTTGAG CGTGATGTCT TCTCACCAAT
CP156633.1    AGACGTGAAT CATCGAATCT TTGAACGCAC ATTGCGCCTT GGGGTATTCC CCAAGGCATG CCTGTTTGAG CGTGATGTCT TCTCACCAAT
CP156631.1    AGACGTGAAT CATCGAATCT TTGAACGCAC ATTGCGCCTT GGGGTATTCC CCAAGGCATG CCTGTTTGAG CGTGATGTCT TCTCACCAAT
CP156630.1    AGACGTGAAT CATCGAATCT TTGAACGCAC ATTGCGCCTT GGGGTATTCC CCAAGGCATG CCTGTTTGAG CGTGATGTCT TCTCACCAAT
CP147453.1    AGACGTGAAT CATCGAATCT TTGAACGCAC ATTGCGCCTT GGGGTATTCC CCAAGGCATG CCTGTTTGAG CGTGATGTCT TCTCACCAAT
CP147467.1    AGACGTGAAT CATCGAATCT TTGAACGCAC ATTGCGCCTT GGGGTATTCC CCAAGGCATG CCTGTTTGAG CGTGATGTCT TCTCACCAAT
```

International Journal of *Molecular Sciences*

MDPI

```
CP147446.1   AGACGTGAAT CATCGAATCT TTGAACGCAC ATTGCGCCTT GGGGTATTCC CCAAGGCATG CCTGTTTGAG CGTGATGTCT TCTCACCAAT
CP147481.1   AGACGTGAAT CATCGAATCT TTGAACGCAC ATTGCGCCTT GGGGTATTCC CCAAGGCATG CCTGTTTGAG CGTGATGTCT TCTCACCAAT
CP147439.1   AGACGTGAAT CATCGAATCT TTGAACGCAC ATTGCGCCTT GGGGTATTCC CCAAGGCATG CCTGTTTGAG CGTGATGTCT TCTCACCAAT
CP147460.1   AGACGTGAAT CATCGAATCT TTGAACGCAC ATTGCGCCTT GGGGTATTCC CCAAGGCATG CCTGTTTGAG CGTGATGTCT TCTCACCAAT
CP147474.1   AGACGTGAAT CATCGAATCT TTGAACGCAC ATTGCGCCTT GGGGTATTCC CCAAGGCATG CCTGTTTGAG CGTGATGTCT TCTCACCAAT
CP147432.1   AGACGTGAAT CATCGAATCT TTGAACGCAC ATTGCGCCTT GGGGTATTCC CCAAGGCATG CCTGTTTGAG CGTGATGTCT TCTCACCAAT
CP126633.2   AGACGTGAAT CATCGAATCT TTGAACGCAC ATTGCGCCTT GGGGTATTCC CCAAGGCATG CCTGTTTGAG CGTGATGTCT TCTCACCAAT
CP126454.2   AGACGTGAAT CATCGAATCT TTGAACGCAC ATTGCGCCTT GGGGTATTCC CCAAGGCATG CCTGTTTGAG CGTGATGTCT TCTCACCAAT
CP126453.2   AGACGTGAAT CATCGAATCT TTGAACGCAC ATTGCGCCTT GGGGTATTCC CCAAGGCATG CCTGTTTGAG CGTGATGTCT TCTCACCAAT
CP126450.2   AGACGTGAAT CATCGAATCT TTGAACGCAC ATTGCGCCTT GGGGTATTCC CCAAGGCATG CCTGTTTGAG CGTGATGTCT TCTCACCAAT
OP626776.1   AGACGTGAAT CATCGAATCT TTGAACGCAC ATTGCGCCTT GGGGTATTCC CCAAGGCATG CCTGTTTGAG CGTGATGTCT TCTCACCAAT
CP133934.1   AGACGTGAAT CATCGAATCT TTGAACGCAC ATTGCGCCTT GGGGTATTCC CCAAGGCATG CCTGTTTGAG CGTGATGTCT TCTCACCAAT
CP133933.1   AGACGTGAAT CATCGAATCT TTGAACGCAC ATTGCGCCTT GGGGTATTCC CCAAGGCATG CCTGTTTGAG CGTGATGTCT TCTCACCAAT
CP133932.1   AGACGTGAAT CATCGAATCT TTGAACGCAC ATTGCGCCTT GGGGTATTCC CCAAGGCATG CCTGTTTGAG CGTGATGTCT TCTCACCAAT
OQ581776.1   AGACGTGAAT CATCGAATCT TTGAACGCAC ATTGCGCCTT GGGGTATTCC CCAAGGCATG CCTGTTTGAG CGTGATGTCT TCTCACCAAT
OQ513465.1   AGACGTGAAT CATCGAATCT TTGAACGCAC ATTGCGCCTT GGGGTATTCC CCAAGGCATG CCTGTTTGAG CGTGATGTCT TCTCACCAAT
OQ513462.1   AGACGTGAAT CATCGAATCT TTGAACGCAC ATTGCGCCTT GGGGTATTCC CCAAGGCATG CCTGTTTGAG CGTGATGTCT TCTCACCAAT
KP326583.1   AGACGTGAAT CATCGAATCT TTGAACGCAC ATTGCGCCTT GGGGTATTCC CCAAGGCATG CCTGTTTGAG CGTGATGTCT TCTCACCAAT
KP131674.1   AGACGTGAAT CATCGAATCT TTGAACGCAC ATTGCGCCTT GGGGTATTCC CCAAGGCATG CCTGTTTGAG CGTGATGTCT TCTCACCAAT
OW988727.1   AGACGTGAAT CATCGAATCT TTGAACGCAC ATTGCGCCTT GGGGTATTCC CCAAGGCATG CCTGTTTGAG CGTGATGTCT TCTCACCAAT
OW988598.1   AGACGTGAAT CATCGAATCT TTGAACGCAC ATTGCGCCTT GGGGTATTCC CCAAGGCATG CCTGTTTGAG CGTGATGTCT TCTCACCAAT
OM530139.1   AGACGTGAAT CATCGAATCT TTGAACGCAC ATTGCGCCTT GGGGTATTCC CCAAGGCATG CCTGTTTGAG CGTGATGTCT TCTCACCAAT
CP076751.1   AGACGTGAAT CATCGAATCT TTGAACGCAC ATTGCGCCTT GGGGTATTCC CCAAGGCATG CCTGTTTGAG CGTGATGTCT TCTCACCAAT
CP077033.1   AGACGTGAAT CATCGAATCT TTGAACGCAC ATTGCGCCTT GGGGTATTCC CCAAGGCATG CCTGTTTGAG CGTGATGTCT TCTCACCAAT
CP076991.1   AGACGTGAAT CATCGAATCT TTGAACGCAC ATTGCGCCTT GGGGTATTCC CCAAGGCATG CCTGTTTGAG CGTGATGTCT TCTCACCAAT
CauITS1-F    ---------- ---------- ---------- ---------- ---------- ---------- ---------- ---------- ----------
CauITS1-R    ---------- ---------- ---------- ---------- ---------- ---------- ---------- ---------- ----------

             ....|....| ....|....| ....|....| ....|....| ....|....| ....|....| ....|....| ....|....| ....|....|
                   275        285        295        305        315        325        335        345        355
MK981227.1   CTTCGCGGTG GCGTTGCATT CACAAAATTA CAGCTTGCAC GAAAAAAATC TACGCTTTTT TTTTCGTTTT GTTGTCGCCT CAAATCAGGT
MK367811.1   CTTCGCGGTG GCGTTGCATT CACAAAATTA CAGCTTGCAC GAAAAAAATC TACGCTTTTT TTTTCGTTTT GTTGTCGCCT CAAATCAGGT
MH071441.1   CTTCGCGGTG GCGTTGCATT CACAAAATTA CAGCTTGCAC GAAAAAAATC TACGCTTTTT TTTTCGTTTT GTTGTCGCCT CAAATCAGGT
MH118269.2   CTTCGCGGTG GCGTTGCATT CACAAAATTA CAGCTTGCAC GAAAAAAATC TACGCTTTTT TTTTCGTTTT GTTGTCGCCT CAAATCAGGT
MH174460.1   CTTCGCGGTG GCGTTGCATT CACAAAATTA CAGCTTGCAC GAAAAAAATC TACGCTTTTT TTTTCGTTTT GTTGTCGCCT CAAATCAGGT
CP163329.1   CTTCGCGGTG GCGTTGCATT CACAAAATTA CAGCTTGCAC GAAAAAAATC TACGCTTTTT TTTTCGTTTT GTTGTCGCCT CAAATCAGGT
CP163322.1   CTTCGCGGTG GCGTTGCATT CACAAAATTA CAGCTTGCAC GAAAAAAATC TACGCTTTTT TTTTCGTTTT GTTGTCGCCT CAAATCAGGT
CP163350.1   CTTCGCGGTG GCGTTGCATT CACAAAATTA CAGCTTGCAC GAAAAAAATC TACGCTTTTT TTTTCGTTTT GTTGTCGCCT CAAATCAGGT
CP163357.1   CTTCGCGGTG GCGTTGCATT CACAAAATTA CAGCTTGCAC GAAAAAAATC TACGCTTTTT TTTTCGTTTT GTTGTCGCCT CAAATCAGGT
CP163336.1   CTTCGCGGTG GCGTTGCATT CACAAAATTA CAGCTTGCAC GAAAAAAATC TACGCTTTTT TTTTCGTTTT GTTGTCGCCT CAAATCAGGT
CP163315.1   CTTCGCGGTG GCGTTGCATT CACAAAATTA CAGCTTGCAC GAAAAAAATC TACGCTTTTT TTTTCGTTTT GTTGTCGCCT CAAATCAGGT
CP163364.1   CTTCGCGGTG GCGTTGCATT CACAAAATTA CAGCTTGCAC GAAAAAAATC TACGCTTTTT TTTTCGTTTT GTTGTCGCCT CAAATCAGGT
```

International Journal of Molecular Sciences

MDPI

CP163343.1	CTTCGCGGT**G**	**GCGTTGCATT**	**CACAAAA**TTA	CAGCTTGCAC	GAAAAAAATC	TACGCTTTTT	TTTTCGTTTT	GTTGTCGCCT	CAAATCAGGT
PP994477.1	CTTCGCGGT**G**	**GCGTTGCATT**	**CACAAAA**TTA	CAGCTTGCAC	GAAAAAAATC	TACGCTTTTT	TTTTCGTTTT	GTTGTCGCCT	CAAATCAGGT
CP157519.1	CTTCGCGGT**G**	**GCGTTGCATT**	**CACAAAA**TTA	CAGCTTGCAC	GAAAAAAATC	TACGCTTTTT	TTTTCGTTTT	GTTGTCGCCT	CAAATCAGGT
CP157517.1	CTTCGCGGT**G**	**GCGTTGCATT**	**CACAAAA**TTA	CAGCTTGCAC	GAAAAAAATC	TACGCTTTTT	TTTTCGTTTT	GTTGTCGCCT	CAAATCAGGT
CP157516.1	CTTCGCGGT**G**	**GCGTTGCATT**	**CACAAAA**TTA	CAGCTTGCAC	GAAAAAAATC	TACGCTTTTT	TTTTCGTTTT	GTTGTCGCCT	CAAATCAGGT
CP157512.1	CTTCGCGGT**G**	**GCGTTGCATT**	**CACAAAA**TTA	CAGCTTGCAC	GAAAAAAATC	TACGCTTTTT	TTTTCGTTTT	GTTGTCGCCT	CAAATCAGGT
CP157510.1	CTTCGCGGT**G**	**GCGTTGCATT**	**CACAAAA**TTA	CAGCTTGCAC	GAAAAAAATC	TACGCTTTTT	TTTTCGTTTT	GTTGTCGCCT	CAAATCAGGT
CP157509.1	CTTCGCGGT**G**	**GCGTTGCATT**	**CACAAAA**TTA	CAGCTTGCAC	GAAAAAAATC	TACGCTTTTT	TTTTCGTTTT	GTTGTCGCCT	CAAATCAGGT
CP156633.1	CTTCGCGGT**G**	**GCGTTGCATT**	**CACAAAA**TTA	CAGCTTGCAC	GAAAAAAATC	TACGCTTTTT	TTTTCGTTTT	GTTGTCGCCT	CAAATCAGGT
CP156631.1	CTTCGCGGT**G**	**GCGTTGCATT**	**CACAAAA**TTA	CAGCTTGCAC	GAAAAAAATC	TACGCTTTTT	TTTTCGTTTT	GTTGTCGCCT	CAAATCAGGT
CP156630.1	CTTCGCGGT**G**	**GCGTTGCATT**	**CACAAAA**TTA	CAGCTTGCAC	GAAAAAAATC	TACGCTTTTT	TTTTCGTTTT	GTTGTCGCCT	CAAATCAGGT
CP147453.1	CTTCGCGGT**G**	**GCGTTGCATT**	**CACAAAA**TTA	CAGCTTGCAC	GAAAAAAATC	TACGCTTTTT	TTTTCGTTTT	GTTGTCGCCT	CAAATCAGGT
CP147467.1	CTTCGCGGT**G**	**GCGTTGCATT**	**CACAAAA**TTA	CAGCTTGCAC	GAAAAAAATC	TACGCTTTTT	TTTTCGTTTT	GTTGTCGCCT	CAAATCAGGT
CP147446.1	CTTCGCGGT**G**	**GCGTTGCATT**	**CACAAAA**TTA	CAGCTTGCAC	GAAAAAAATC	TACGCTTTTT	TTTTCGTTTT	GTTGTCGCCT	CAAATCAGGT
CP147481.1	CTTCGCGGT**G**	**GCGTTGCATT**	**CACAAAA**TTA	CAGCTTGCAC	GAAAAAAATC	TACGCTTTTT	TTTTCGTTTT	GTTGTCGCCT	CAAATCAGGT
CP147439.1	CTTCGCGGT**G**	**GCGTTGCATT**	**CACAAAA**TTA	CAGCTTGCAC	GAAAAAAATC	TACGCTTTTT	TTTTCGTTTT	GTTGTCGCCT	CAAATCAGGT
CP147460.1	CTTCGCGGT**G**	**GCGTTGCATT**	**CACAAAA**TTA	CAGCTTGCAC	GAAAAAAATC	TACGCTTTTT	TTTTCGTTTT	GTTGTCGCCT	CAAATCAGGT
CP147474.1	CTTCGCGGT**G**	**GCGTTGCATT**	**CACAAAA**TTA	CAGCTTGCAC	GAAAAAAATC	TACGCTTTTT	TTTTCGTTTT	GTTGTCGCCT	CAAATCAGGT
CP147432.1	CTTCGCGGT**G**	**GCGTTGCATT**	**CACAAAA**TTA	CAGCTTGCAC	GAAAAAAATC	TACGCTTTTT	TTTTCGTTTT	GTTGTCGCCT	CAAATCAGGT
CP126633.2	CTTCGCGGT**G**	**GCGTTGCATT**	**CACAAAA**TTA	CAGCTTGCAC	GAAAAAAATC	TACGCTTTTT	TTTTCGTTTT	GTTGTCGCCT	CAAATCAGGT
CP126454.2	CTTCGCGGT**G**	**GCGTTGCATT**	**CACAAAA**TTA	CAGCTTGCAC	GAAAAAAATC	TACGCTTTTT	TTTTCGTTTT	GTTGTCGCCT	CAAATCAGGT
CP126453.2	CTTCGCGGT**G**	**GCGTTGCATT**	**CACAAAA**TTA	CAGCTTGCAC	GAAAAAAATC	TACGCTTTTT	TTTTCGTTTT	GTTGTCGCCT	CAAATCAGGT
CP126450.2	CTTCGCGGT**G**	**GCGTTGCATT**	**CACAAAA**TTA	CAGCTTGCAC	GAAAAAAATC	TACGCTTTTT	TTTTCGTTTT	GTTGTCGCCT	CAAATCAGGT
OP626776.1	CTTCGCGGT**G**	**GCGTTGCATT**	**CACAAAA**TTA	CAGCTTGCAC	GAAAAAAATC	TACGCTTTTT	TTTTCGTTTT	GTTGTCGCCT	CAAATCAGGT
CP133934.1	CTTCGCGGT**G**	**GCGTTGCATT**	**CACAAAA**TTA	CAGCTTGCAC	GAAAAAAATC	TACGCTTTTT	TTTTCGTTTT	GTTGTCGCCT	CAAATCAGGT
CP133933.1	CTTCGCGGT**G**	**GCGTTGCATT**	**CACAAAA**TTA	CAGCTTGCAC	GAAAAAAATC	TACGCTTTTT	TTTTCGTTTT	GTTGTCGCCT	CAAATCAGGT
CP133932.1	CTTCGCGGT**G**	**GCGTTGCATT**	**CACAAAA**TTA	CAGCTTGCAC	GAAAAAAATC	TACGCTTTTT	TTTTCGTTTT	GTTGTCGCCT	CAAATCAGGT
OQ581776.1	CTTCGCGGT**G**	**GCGTTGCATT**	**CACAAAA**TTA	CAGCTTGCAC	GAAAAAAATC	TACGCTTTTT	TTTTCGTTTT	GTTGTCGCCT	CAAATCAGGT
OQ513465.1	CTTCGCGGT**G**	**GCGTTGCATT**	**CACAAAA**TTA	CAGCTTGCAC	GAAAAAAATC	TACGCTTTTT	TTTTCGTTTT	GTTGTCGCCT	CAAATCAGGT
OQ513462.1	CTTCGCGGT**G**	**GCGTTGCATT**	**CACAAAA**TTA	CAGCTTGCAC	GAAAAAAATC	TACGCTTTTT	TTTTCGTTTT	GTTGTCGCCT	CAAATCAGGT
KP326583.1	CTTCGCGGT**G**	**GCGTTGCATT**	**CACAAAA**TTA	CAGCTTGCAC	GAAAAAAATC	TACGCTTTTT	TTTTCGTTTT	GTTGTCGCCT	CAAATCAGGT
KP131674.1	CTTCGCGGT**G**	**GCGTTGCATT**	**CACAAAA**TTA	CAGCTTGCAC	GAAAAAAATC	TACGCTTTTT	TTTTCGTTTT	GTTGTCGCCT	CAAATCAGGT
OW988727.1	CTTCGCGGT**G**	**GCGTTGCATT**	**CACAAAA**TTA	CAGCTTGCAC	GAAAAAAATC	TACGCTTTTT	TTTTCGTTTT	GTTGTCGCCT	CAAATCAGGT
OW988598.1	CTTCGCGGT**G**	**GCGTTGCATT**	**CACAAAA**TTA	CAGCTTGCAC	GAAAAAAATC	TACGCTTTTT	TTTTCGTTTT	GTTGTCGCCT	CAAATCAGGT
OM530139.1	CTTCGCGGT**G**	**GCGTTGCATT**	**CACAAAA**TTA	CAGCTTGCAC	GAAAAAAATC	TACGCTTTTT	TTTTCGTTTT	GTTGTCGCCT	CAAATCAGGT
CP076751.1	CTTCGCGGT**G**	**GCGTTGCATT**	**CACAAAA**TTA	CAGCTTGCAC	GAAAAAAATC	TACGCTTTTT	TTTTCGTTTT	GTTGTCGCCT	CAAATCAGGT
CP077033.1	CTTCGCGGT**G**	**GCGTTGCATT**	**CACAAAA**TTA	CAGCTTGCAC	GAAAAAAATC	TACGCTTTTT	TTTTCGTTTT	GTTGTCGCCT	CAAATCAGGT
CP076991.1	CTTCGCGGT**G**	**GCGTTGCATT**	**CACAAAA**TTA	CAGCTTGCAC	GAAAAAAATC	TACGCTTTTT	TTTTCGTTTT	GTTGTCGCCT	CAAATCAGGT
CauITS1-F	----------	----------	----------	----------	----------	----------	----------	----------	----------
CauITS1-R	---------**G**	**GCGTTGCATT**	**CACAAAA**---	----------	----------	----------	----------	----------	----------

International Journal of Molecular Sciences

MDPI

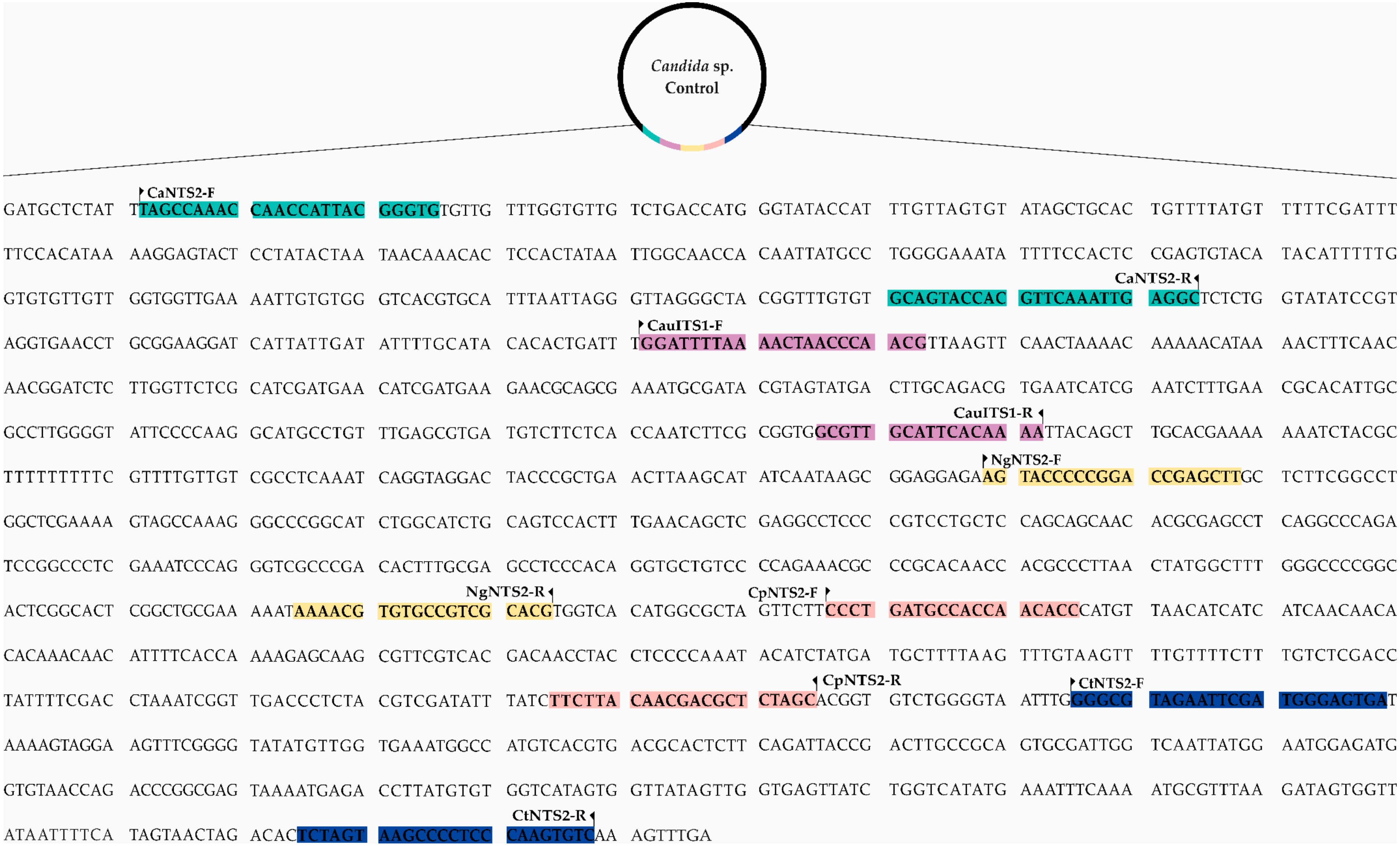

Figure S1: Construction of the positive control (using the pUC57 plasmid) carrying the consensus sequences of the target genes used in melting-curve based qPCR. CaNTS2 (*Candida albicans* primer); CauITS1 (*Candida auris* primer); NgNTS2 (*Nakaseomyces glabratus* primer); CpNTS (*Candida parapsilosis* primer); CtNTS2 (*Candida tropicalis* primer).

International Journal of
Molecular Sciences

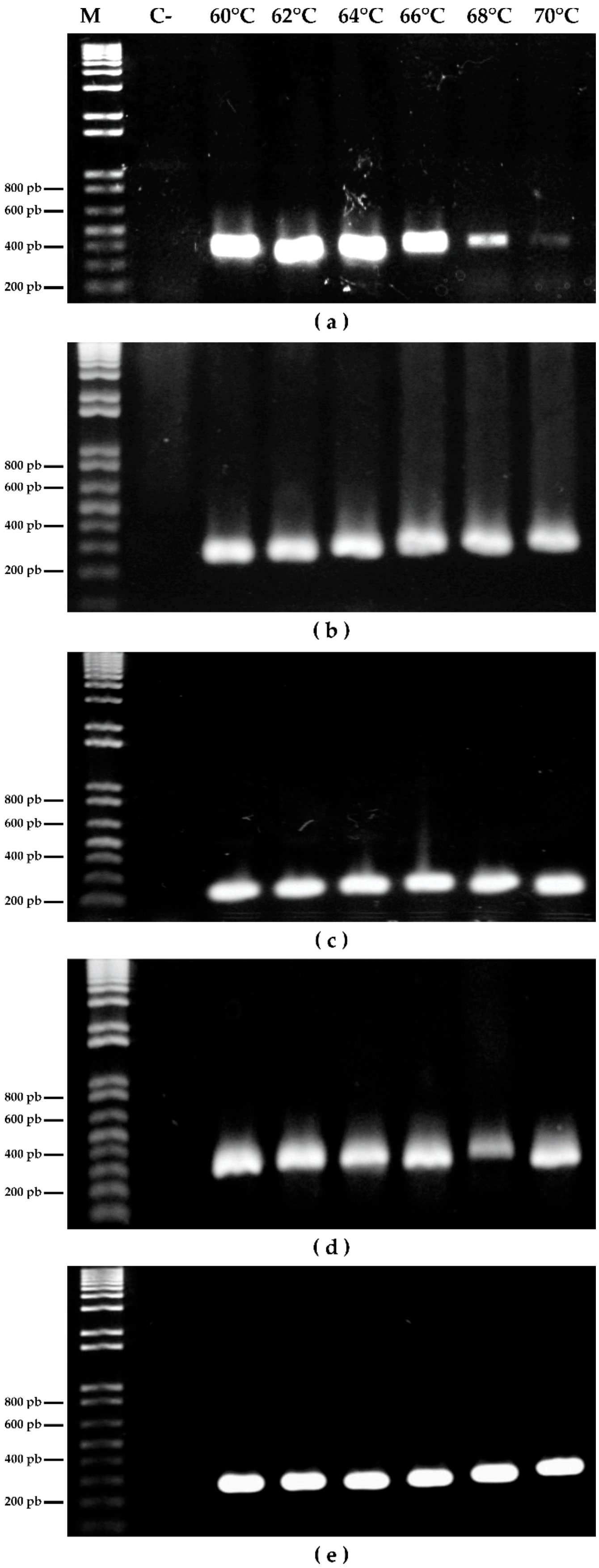

Figure S2: Gradient PCR optimization of species-specific fungal amplification. Gradient PCR was performed to determine the optimal annealing temperatures (ranging from 60 °C to 70 °C, as indicated above first agarose gel) for the amplification of five fungal targets. Fungal genomic DNA samples included: *Candida albicans* **a**), *Candida auris* **b**), *Candida parapsilosis* **c**), *Candida tropicalis* **d**), and *Nakaseomyces glabratus* **e**), and 1µM of each primer. Lane M contains the DNA molecular weight marker (1 kb plus DNA ladder)

International Journal of
Molecular Sciences

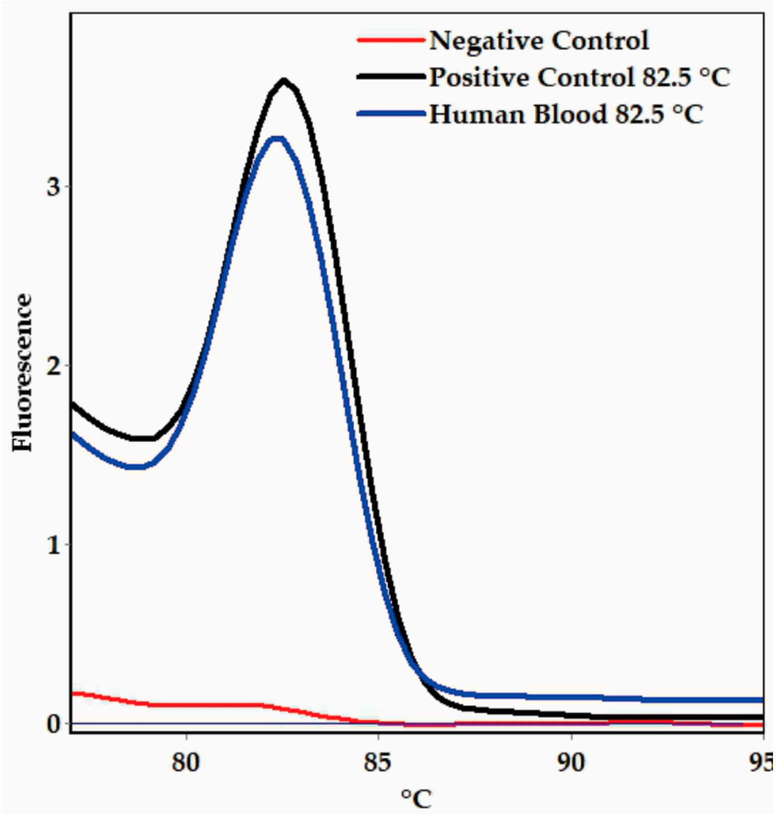

Figure S3: Melting curve analysis of human RNase P amplification. Melting curves show the specific melting temperature (Tm) peaks for PCR-amplified human tRNA-processing ribonuclease P (RNase P).
